# Nitrogen-Doped Graphene Materials with High Electrical Conductivity Produced by Electrochemical Exfoliation of Graphite Foil

**DOI:** 10.3390/nano14010123

**Published:** 2024-01-04

**Authors:** Hela Kammoun, Benjamin D. Ossonon, Ana C. Tavares

**Affiliations:** Centre Énergie Matériaux Télécommunications, Institut National de la Recherche Scientifique, 1650 Boulevard Lionel-Boulet, Varennes, QC J3X 1P7, Canada; hela.kammoun@inrs.ca (H.K.); benjamin.ossonon@inrs.ca (B.D.O.)

**Keywords:** electrolyte effect, primary and secondary amine groups, substitutional doping, reduced graphene oxide, graphitic nitrogen, hydroxyl groups, electrical conductivity

## Abstract

Nitrogen-doped graphene-based materials are of utmost importance in sensing and energy conversion devices due to their unique physicochemical properties. However, the presence of defects such as pyrrolic nitrogen and oxygenated functional groups reduces their electrical conductivity. Herein, a two-step approach based on the electrochemical exfoliation of graphite foils in aqueous mixed electrolytes followed by thermal reduction at 900 °C is used to prepare high-quality few layers of N-doped graphene-based materials. The exfoliations were conducted in 0.1 M (NH_4_)_2_SO_4_ or H_2_SO_4_ and HNO_3_ (5 mM or 0.1 M) electrolytes mixtures and the HNO_3_ vol% varied. Chemical analysis demonstrated that the as-prepared graphene oxides contain nitro and amine groups. Thermal reduction is needed for substitutional N-doping. Nitrogen and oxygen surface concentrations vary between 0.23–0.96% and 3–8%, respectively. Exfoliation in (NH_4_)_2_SO_4_ and/or 5 mM HNO_3_ favors the formation of pyridinic-N (10–40% of the total N), whereas 1 M HNO_3_ favors the formation of graphitic-N (≈60%). The electrical conductivity ranges between 166–2705 Scm^−1^. Raman spectroscopy revealed a low density of defects (I_D_/I_G_ ratio between 0.1 and 0.7) and that most samples are composed of mono-to-bilayer graphene-based materials (I_G_/I_2D_ integrated intensities ratio). Structural and compositional stability of selected samples after storage in air for three months is demonstrated. These results confirm the high quality of the synthesized undoped and N-doped graphene-type materials.

## 1. Introduction

During the last few decades, graphene-based compounds have emerged as promising materials for a variety of applications because of their appealing properties such as high thermal and electronic conductivities, large surface area, easiness of synthesis, as well as tunable structural, electronic and optical properties through chemical doping and surface functionalization [1,2,3]. Nitrogen is a popular substitutional dopant because it can easily replace carbon atoms in the graphitic network in the form of graphitic, pyridinic and pyrrolic nitrogen [4]. Doping with N changes the electronic structure of graphene by shifting the Fermi level away from the Dirac point [3]. In addition, nitrogen has five valence electrons and higher electronegativity (3.44) compared to carbon (2.55). Thus, N-doping induces polarization in the sp^2^ carbon network and modifies the local charge of the graphitic layer. Thereby, the N-species and the adjacent carbons can act as the active sites in a variety of processes [5], and directly affect the performance of graphene-based sensing devices because their sensitivity is governed by the exchange of electrons between the target molecule and the graphene backbone [2,6]. Likewise, N-doping influences the phonon dynamics in graphene-based materials [7,8]. N-doped graphene materials have been combined with semiconductors to fabricate photoactive materials [9] or in p-n junctions for photodetectors [10]. They have also been used in coatings to prevent corrosion [11]. Additionally, they can enhance cell viability thanks to their biocompatibility [12]. They have also been used in anodes of lithium-ion batteries, with improved reversible capacity and cyclability [13,14,15]. Disorder and excess of local negative charge in the graphene layers offer more insertion sites for Li^+^ ions and are among the various effects induced by substitutional N-doping leading to better performance. However, the role of the N-species is different: pyridinic N allows for more Li^+^ storage, and pyrrolic N enhances the surface transport of Li^+^ ions [15]. N-doped graphene has also been investigated as electrode material for supercapacitors [16,17,18]. Similarly, N-doping increases the electrostatic interaction between the graphene layers and the ions in the electrolyte, and so the specific capacitance and life cycling of the electrodes. Additionally, N-doped graphene-based materials are of most importance for the development of Pt group metal-free catalysts [19] and metal-free carbon-based catalysts [20] for the oxygen reduction reaction for fuel cells, and in-situ electrogeneration of H_2_O_2_ [21]. However, the structural factors (content and type of N-functionalities, carbon adjacent to the N-atom, fraction of sp^2^ domains, interface between sp^2^/sp^3^ domains, topological defects) leading to an enhancement of the activity and selectivity (H_2_O vs. H_2_O_2_) are still under debate [20,22,23,24,25,26].

Two approaches are usually followed to prepare N-doped graphene materials: direct synthesis or post-treatment of graphene-type materials such as graphene oxide [1,18,27]. Direct synthesis is often conducted by physical methods such as chemical vapor deposition using C_2_H_2_/NH_4_, CH_4_/NH_3_ or even acetonitrile or arc discharge between graphite electrodes in the presence of H_2_, NH_3_ or pyridine vapor. However, these methods are complex, energy-consuming, require sophisticated equipment, and only the top layer is doped with nitrogen. In post-treatment physical methods such as plasma treatment, graphite oxide is exposed to a nitrogen plasma and carbon atoms are replaced by nitrogen ones [28]. However, oxygenated species are formed during the process, and the oxygen content can be as high as 30 at% [29]. Post-synthesis of N-doped graphene materials can also be done by high-temperature thermal annealing of graphene oxide (GO) or pristine graphene in the presence of nitrogen source (N_2_, NH_3_ gases) [1,30] or by mixing with N-sources such as melamine [31], nitric acid [32], uric acid [33], and mixtures of hydrazine and tetrachloromethane [34]. The N-doping level can be as high as 16.4% depending on the nature and concentration of the nitrogen source, as well as the type of GO material.

Electrochemical exfoliation of bulk materials is a versatile method to prepare 2D materials with tunable properties [35,36]. Electrochemical exfoliation of graphite foils, rods and plates is perhaps a green, cost-effective and yet simple approach to obtaining low-defect graphene materials. It uses simple setups and can be easily scaled up for large-scale production of graphene materials [35,36,37,38,39,40]. Electrochemical exfoliation of graphite occurs through three stages. The edges and grain boundaries of graphite are attacked by oxidizing species (OH^−^, OH^•^, O^•^), followed by the intercalation of anions (anodic exfoliation if positive bias is applied [36,41], cations (cathodic exfoliation if negative bias is applied [42,43]), and water molecules between the graphitic layers. The intercalation step leads to graphite expansion and an increase in the interlayer distance. The evolution of oxygen and other gases during this stage further increases the interlayer distance, leading to the exfoliation of the graphene oxide sheets [44,45]. Graphene-based materials can also be prepared by bipolar exfoliation, a process where a voltage difference is applied between two external electrodes to polarize in a “wireless” manner a graphite source (rod, flakes) present in the electrolyte [46,47]. A post-treatment step is usually needed to obtain reduced graphene oxide, like thermal treatment above 600 °C [38] or an alternating current during exfoliation [48]. The method is very versatile, and parameters such as the applied voltage, the exfoliation duration, and the composition of the electrolytes (solutions of strong acids, inorganic salts or ionic liquids) can be manipulated to tailor the graphene oxide flakes’ lateral size and morphology, oxidation degree, extent of the defects, as well as the presence and content of functional groups and transition metal oxides nanoparticles on their surface [38,44,45,49,50,51,52,53].

Doping of electrochemically exfoliated graphene oxide by heat treatment under NH_3_ atmosphere was reported previously [30]. Exfoliation of graphite in electrolytes containing N-moieties has also been explored to prepare N-doped graphene oxide materials. Examples include the electrochemical exfoliation of graphite foils tetra-n-butylammonium bisulfate followed by the reduction of the graphene oxide using alternating current [48]; electrochemical exfoliation of graphite rod in a protic ionic liquid ethylammonium nitrate [54]; exfoliation of graphite rod in a mixture of glycine and ammonia solution [55]; the electrochemical exfoliation of highly oriented pyrolytic graphite (HOPG) or graphite rod in ethylammonium nitrate [54]; or the exfoliation of graphite rod in 0.1 M (NH_4_)_2_SO_4_ electrolyte [44]. In some of these examples, the nitrogen content can be as high as 2.4 at% [54], 3.4 at% [56], or 6.05 at% [55], but no thermal reduction was applied to these materials.

N-doping, oxygenated, and other functional groups disrupt the carbon sp^2^ domains of the graphitic network and localize the π electrons, resulting in low conductivity [25,57]. The electrical conductivity of N-doped REGO depends on the synthesis and reduction methods. It has been reported that the electrical conductivity of N-doped graphene synthesized by the Hummer’s method and reduced under N_2_ is 56.8 Scm^−1^ [22], but when reduced under NH_3_ at 800 °C it has an electrical conductivity of 316 Scm^−1^ [58]. Hydrothermal synthesis in (NH_4_)_2_CO_3_/GO (1:100) produces N-RGO with an electrical conductivity of 800 Scm^−1^ [59]. Alternating current electrochemical exfoliation of graphite foil in tetrabutyl ammonium—H_2_SO_4_ leads to N-REGO with an electrical conductivity of 640 Scm^−1^ [48]. The number and extent of the sp^2^ domains determine the electrical conductivity of the graphene materials [22,60], which is critical for their application in sensing, energy storage and conversion. Thus, optimization of the electrochemical exfoliation of the graphite process is necessary to obtain graphene-based materials with tunable physical-chemical properties and high electrical conductivity. Consequently, it is important to study systematically the influence of the exfoliating medium on the N-doping level, type of N-species, structural defects, and electrical conductivity of N-doped graphene materials.

In this work, the electrochemical exfoliation of graphite foils was first conducted in 0.1 M (NH_4_)_2_SO_4_ as a function of time and then in mixed electrolytes composed of 0.1 M (NH_4_)_2_SO_4_ + (5 mM or 1 M) HNO_3_ and 0.1 M H_2_SO_4_ + 1 M HNO_3_, where the vol% of HNO_3_ was systematically varied between 0 and 100%. On the one hand, (NH_4_)_2_SO_4_ and H_2_SO_4_ were selected for their high exfoliation efficiency due to the facile intercalation of HSO_4_^−^/SO_4_^2−^ anions between the graphitic layers [44,61]. On the other hand, both (NH_4_)_2_SO_4_ and HNO_3_ [62] were used as nitrogen sources for substitutional doping of the graphene material after thermal treatment at 900 °C.

## 2. Materials and Methods

### 2.1. Materials

Graphite foil (0.5 mm thick, 99.8%) was purchased from Alfa Aesar (Haverhill, MA, USA), sulfuric acid (H_2_SO_4_, 95.0–98.0%) from Sigma-Aldrich (St. Louis, MO, USA), ammonium sulfate ((NH_4_)_2_SO_4_ > 99%) was purchased from Fisher Chemicals (Fair Lawn, NJ, USA), and nitric acid (HNO_3_, 63–70%) from Acros Organics (Fair Lawn, NJ, USA).

### 2.2. Synthesis of N-Doped Reduced Graphene Oxide

A one-compartment, cylindrical, glass electrochemical cell was equipped with a graphite foil (7.5 cm × 2.5 cm × 0.05 cm) working as an anode and connected to the positive end of a DC Power supply (HY3005F-3, from Dr. Meter (Shanghai, China)), and a Pt mesh (7 cm × 2 cm) working as cathode and connected to the negative end of the power supply. Both electrodes were immersed in 200 mL electrolyte solution, keeping a fixed distance of 6 cm between them. The electrochemical exfoliation of graphite was conducted in three different mixed electrolytes: 0.1 M (NH_4_)_2_SO_4_ + 5 mM HNO_3_, 0.1 M (NH_4_)_2_SO_4_ + 1 M HNO_3_ and 0.1 M H_2_SO_4_ + 1 M HNO_3_. The volumetric proportions between two single electrolytes were varied (0, 10, 20, 50, 75, 100 vol%), and the mixtures were sonicated for 5 min before experiments. The exfoliations were carried out by applying a potential difference of 8 V for a total duration of 1 h, except for 100 vol% 1 M HNO_3_ which proceeded till the spontaneous end of the reaction (15 min). The exfoliation in 100 vol% 0.1 M (NH_4_)_2_SO_4_ was extended to 6 h and 12 h.

The exfoliated graphene oxide (EGO) was vacuum filtered using MF-Millipore membranes with 0.22 µm pore size washed thoroughly with deionized water (18.2 MΩ·cm). The EGO powders were then dispersed in water (0.01 mg·mL^−1^) and sonicated for 30 min. Next, 20 mL of the dispersions were used to prepare 3.5 cm diameter and 1 µm thickness EGO films by vacuum filtration using a MF-Millipore membrane with a 0.1 µm pore size and dried at 100 °C under vacuum for 24 h. Finally, reduced EGO (REGO) films were obtained after thermal annealing in an OTF-1200X-III high-temperature tube furnace at 900 °C under Argon flow. These films were used for Raman and XPS spectroscopies and for electrical conductivity measurements.

### 2.3. Physicochemical Characterization

Raman spectra were recorded on a Raman microscope (Renishaw, inVia) using a laser source with a wavelength of 532 nm. The laser beam was focused on the sample with a spot size of 2 cm in diameter. The spectra were acquired in static mode over 120 to 4500 cm^−1^ with an exposure duration of 10 s and for a total of 3 scans. At least 5 spectra were recorded for each sample. The deconvolution of the 2D peak was done by fitting with a Lorentzian function.

X-ray photoelectron spectroscopy (XPS) analysis of all prepared EGO and REGO samples was carried out in a VG Escalab 200i-XL spectrometer using a Twin Anode Al X-Ray Source operated at 15 kV and 20 mA, a hemispherical analyzer (pass energy = 20 eV) and a multi-channel detector. Base pressure inside the spectrometer was less than 7 × 10^−10^ torr during analysis. The binding energy of C1s (284.4 eV) was used as an internal standard. The core-level spectra were fitted using Lorentzian and Gaussian curves with a Shirley-type background using Casa XPS software v2.3.24. The surface at% of C, O and N were determined by integrating the entire 1 s core level spectra, and the areas were normalized by the appropriate atomic sensitivity factors.

Fourier Transform Infrared (FT-IR) spectra of the powder samples were recorded in the region 3750–750 cm^−1^ on a Nicolet/Smart ITR FT-IR spectrophotometer using OMNIC software v8.3. Each sample was scanned 64 times with a resolution of 4.0 cm^−1^. Thermogravimetric analysis (TGA) combined with mass spectroscopy (MS) was performed on TA Instruments, TGA Q500/Discovery MS. Samples were placed in a Pt pan and heated from 30 to 900 °C with a temperature ramp of 5 °C/min, under flowing air. The Differential thermogravimetric (DTG) plots were obtained after processing TGA results with TRIOS.

The resistivity of free-standing films (1 µm thickness) was measured at room temperature using a 4-point probe method with a Keithley 6220 DC precision current source. The electrical conductivity was calculated using Ohm’s Law:

(1) $$\sigma =\frac{1}{\rho }=\frac{l}{R\times A}\;,$$

where σ is the electrical conductivity (Scm^−1^), ρ the electrical resistivity (Ω·cm), R the resistance (Ω), l the distance between two internal probes (cm), and A is the cross-section area of the film (cm^2^).

Morphological features were visualized using Scanning Electron Microscopy (SEM) by Tescan Vega 3 microscope operating at 20.0 keV incident energy.

The stability and purity of selected REGO samples as prepared and exposed to air for three months were investigated by X-ray diffraction (XRD), Transmission electronic microscopy (TEM) and XPS. The XRD studies were conducted on a Bruker D8 X-ray diffractometer with Cu-Kα radiation (λ = 1.54178 Å). The diffractograms were acquired using a step size of 0.04° and a step time of 2 s. A Talos F200X G2 electron microscope was used for TEM analysis. A PHI’s Quantes instrument equipped with a monochromatic Al (1486.6 eV) source was used for the XPS analysis of these samples. Survey spectra were recorded with a pass energy of 280 eV and a step of 1 eV. A pass energy and step size of 55 eV and 0.1 eV, respectively, were used for high-resolution measurements. The acquisition time of the various core-level spectra was adjusted to obtain a good signal-to-noise ratio. Charge compensation was maintained by combining a low-intensity ion beam with a low-energy electron beam. In addition, the main chamber pressure was kept below 10^−^^6^ Pa during the measurements. Analysis of the XPS spectra was conducted, as explained above.

## 3. Discussion

### 3.1. Exfoliation of Graphite Foil in (NH_4_)_2_SO_4_

It was previously reported that no N-doping of graphene-type materials occurred during the electrochemical exfoliation of graphite foil in the presence of (NH_4_)_2_SO_4_ [56]. Instead, N-doping or functionalization with amine/amide functional groups was possible when a graphite rod [56] or HOPG [63] was used. In both cases, the exfoliation time is within 10 to 30 min. Therefore, in the first part of this work, the exfoliation of the graphite foil in (NH_4_)_2_SO_4_ is investigated in detail and conducted for 1, 6 and 12 h. The as-prepared materials (EGO) and those obtained after thermal reduction (REGO) were characterized by Raman, XPS, FTIR and TGA-MS.

**Figure 1:**
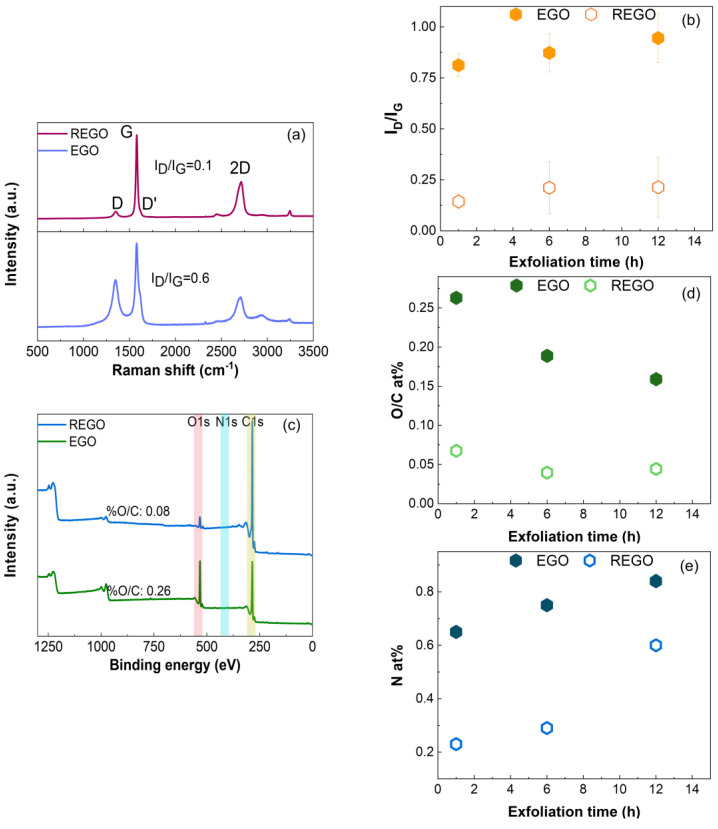
(**a**) Raman and (**c**) XPS survey spectra of EGO and REGO obtained by electrochemical exfoliation of graphite in 0.1 M (NH_4_)_2_SO_4_ electrolyte for 1 h before (EGO) and after reduction at 900 °C (REGO). Variation of (**b**) I_D_/I_G_, (**d**) O/C at% and (**e**) N at% as a function of the exfoliation time in 0.1 M (NH_4_)_2_SO_4_ electrolyte, before (closed symbols) and after reduction at 900 °C (open symbols).

Figure 1a presents the Raman spectra for EGO and REGO obtained after 1 h of exfoliation. The typical G, D and 2D bands of graphene-type materials are visible between 800–3000 cm^−1^. The G band is associated with the graphitic structure through an in-plane C sp^2^ vibration, and the D band is related to the disorder concentration and represents the out-of-plane variation of sp^2^ carbon rings active near edges and defects. The 2D band is the second order of the D band and is related to the number of graphene layers [64,65]. The Raman spectrum of the graphite foil was recorded and used as a reference (Figure S1a). As illustrated in Figure 1a, the intensity of the D band is always lower than that of the G band, confirming the high quality of the graphene-type materials obtained by electrochemical exfoliation in (NH_4_)_2_SO_4_, even before a thermal reduction. The I_D_/I_G_ ratio, which is a measure of the density of defects [65], increases from 0.78 to 0.95 (0.14 to 0.21) for the EGO (REGO) materials as the exfoliation time increases from 1 h to 12 h, Figure 1b. As expected, the I_D_/I_G_ ratio decreased significantly after the thermal treatment [38,39,44]. According to the deconvolution of the 2D band [64,66,67,68] and to the I_G_/I_2D_ integrated intensity ratio [69,70,71], the exfoliation of graphite foil in 0.1 M (NH_4_)_2_SO_4_ produces mostly bilayer-reduced graphene oxide flakes, Figure S1 and Table S1.

**Figure 2:**
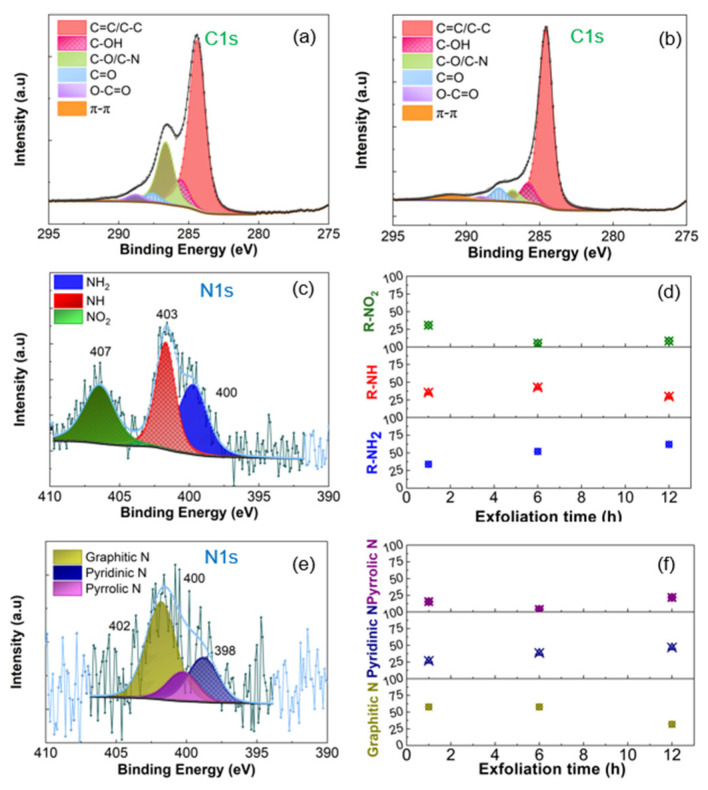
C 1s and N 1s core level spectra of (**a**,**c**) EGO and (**b**,**e**) REGO. Effect of exfoliation time on the type of N-species (**d**) before and (**f**) after thermal reduction at 900 °C for 1 h. The electrochemical exfoliation of the graphite foil was performed in 0.1 M (NH_4_)_2_SO_4_.

Figure 1c presents the survey XPS spectra for EGO and REGO obtained after 1 h of exfoliation. The characteristic C 1s and O 1s lines are clearly visible. The N 1s peak is hardly seen in the survey spectra, but the presence of N-species was confirmed through the core level spectra (see Figure 2 below). Interestingly, the O/C at% ratio of EGO decreases from 0.26 to 0.16 as the exfoliation time increases from 1 h to 12 h, Figure 1d. This decrease is complemented by an increase of the amount of nitrogen species from 0.65% (1 h) to 0.85% (12 h), Figure 1e. An increase in the exfoliation time allows for a sustained formation of oxidizing species at the vicinity of the graphite electrode and nucleophilic attack of the graphite foil by these species [44,72]. In this work, the exfoliation time is at least one hour. Thus, the freshly formed EGO flakes dispersed in the electrolyte most likely endure a nucleophilic attack by the OH^−^ anions, HO^·^ and O^·^ radicals. On the one hand, the oxygenated functional groups are key intermediates in the functionalization of graphene materials by N-moieties [73,74]. On the other hand, a longer exposure of the EGO flakes to the electrolyte allows for more oxygenated functional groups to react with (NH_4_)^+^/NO_2_^−^ ions (see below). The thermal reduction decreases the oxygenated and nitrogen species by a factor of 3, which is unsurprising since it was carried out at 900 ℃ [75]. A similar trend of N% vs. exfoliation time was observed before and after thermal reduction, which suggests that the N content could be further increased by extending the exfoliation time in (NH_4_)_2_SO_4_.

The C 1s and N 1s core level spectra were analyzed to determine the surface chemistry of the as exfoliated and thermally reduced materials. The C 1s core level spectra of EGO and REGO obtained after 1 h of exfoliation, Figure 2a,b, respectively, were deconvoluted into six peaks identified as sp^2^ and sp^3^ carbon at 284.4 eV, C-OH at 285.6 eV, C-O/C-N at 286.6 eV, C=O at 287.5 eV, O-C=O at 288.8 eV and π-π stacking at 290.5 eV [38]. The N 1s spectrum of the as-prepared sample, Figure 2c, was deconvoluted into 3 peaks ascribed to primary amine groups (C-NH_2_) at 399.6 eV, secondary amine groups (C-NH) at 401.6 eV and nitrite groups (C-NO_2_) at 406.4 eV [76,77]. Attribution of these species to the amine groups and not to pyrrolic (400.2 eV) nor pyridinic N (398.9 eV) was supported by additional FTIR and TGA-MS analysis. For example, the FTIR spectra of EGO show the presence of characteristic bands of amine groups at 780 and 3520 cm^−1^, as well as the one attributed to nitro groups at 1330 and 1416 cm^−1^ in Figure S2a. Fragments with *m/z* = 17 were detected using TGA-MS analysis, Figure S3. Detailed analysis of the FTIR and TGA/DTG-MS data are included in the Supporting information [73,78,79,80,81,82,83,84,85,86,87,88,89,90]. As previously shown [63,84] and confirmed in this work, XPS alone is insufficient to determine the composition of graphene oxide materials modified with N-moieties.

Figure 2d shows the variation of the relative concentration of nitrogen species with the exfoliation time before the thermal treatment at 900 °C. As time increases, the content of primary amines increases from 34% to about 62% of secondary amines decreases slightly (from 36% to 29%), and the amount of NO_2_ groups decreases significantly (from ca. 31% to about 8%). The ${NH}_{4}^{+}$ ions are the only source of the three N species and the variation of their relative content over time can be explained by the reactions described in Equations (2)–(6).


(2) $$2{NH}_{4}^{+}+{3O}_{2}\to {2NO}_{2}^{-}+{2H}_{2}O+{4H}^{+}$$

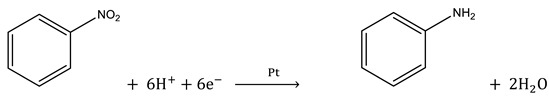
(3)



(4) $$2{NH}_{4}^{+}+{OH}^{-}\to {3NH}_{3}+H_{2}O$$

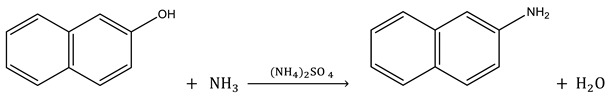
(5)




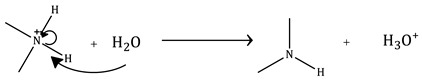
(6)


The formation of NO_2_ groups results from the oxidation of the ammonium ions. On one hand, these cations can be adsorbed on the surface of freshly exfoliated graphene oxide sheets, which are then oxidized to nitrite anions by O_2_ molecules (Equation (2)) formed during water oxidation. On the other hand, freshly formed nitrite anions can adsorb on the active defect sites created by hydroxyl and carbonyl groups on the EGO sheets [91]. Afterward, primary amines are formed by the reduction of nitro groups, according to Equation (3), catalyzed by the platinum counter electrode [92,93,94]. Additionally, primary amines can be formed by aromatic nucleophilic substitution on naphthol (hydroxyl groups in EGO) in the presence of NH_3_ (Equation (4)) formed according to the Butcherer reaction (Equation (5)) [95]. Secondary amines are converted to primary amines, according to Equation (6). Thus, the decrease in concentration of nitro groups with the exfoliation time is explained by their conversion to primary amines (Equation (3)).

After thermal reduction at 900 °C, nitrogen is introduced into the graphene network, evidenced by the presence of graphitic nitrogen at 401.8 eV, pyrrolic nitrogen at 400.2 eV and pyridinic nitrogen at 398.8 eV [96], Figure 2e. The C 1s and N 1s of EGO and REGO obtained after 6 and 12 h of exfoliation have the same features, differing only by the relative amount of the N-species. An increase in the exfoliation time from 1 h to 12 h decreases the percentage of graphitic nitrogen from 58% to 32%, Figure 2f. However, the content of pyridinic nitrogen increases from 27% to 47%, and pyrrolic nitrogen amounts from 15% to 22%. Indeed, as the exfoliation time increases, more nitrogen functional groups on the edges and defects are created, favoring substitutional doping [74]. The presence of pyridinic, pyrrolic and graphitic nitrogen is also confirmed by FTIR analysis with characteristic bands at 1192 and 2157 for pyridinic nitrogen, 1005 and 1107 for pyrrolic nitrogen and 1360–1400 cm^−1^ for graphitic nitrogen, Figure S2b. The mechanisms of formation of pyridinic, pyrrolic, and graphitic nitrogen during thermal treatment of graphene oxide are not fully known and continue to be a subject of investigation. Nevertheless, it was hypothesized that the formation of pyridinic nitrogen is thought to occur through the nucleophilic attack of nitrogen-containing species on the epoxide, hydroxyl, and carboxyl functional groups [73,74]. Pyrrolic nitrogen formation is also believed to occur through the nucleophilic attack of nitrogen-containing species on the carbonyl functional groups of GO [73,74]. Finally, the formation of graphitic nitrogen is thought to occur through a substitution reaction in which nitrogen free radicals react with the carbon atoms in the graphene lattice [4].

### 3.2. Exfoliation of Graphite Foil in Mixed Electrolytes

The exfoliation of graphite foils in (NH_4_)_2_SO_4_ electrolyte solution followed by thermal reduction at 900 °C allows to produce high quality (very low I_D_/I_G_ and O at%) of N-doped reduced graphene oxide materials, Figure 1. Furthermore, the defects fraction is low even when exfoliation is extended to 12 h. However, the N-content is low (between 0.2 to 0.6%) due to the low oxygen content on the as-prepared EGOs and the high temperature used to reduce them [97]. Therefore, in an attempt to increase the N-content and to evaluate the impact of the composition of the electrolyte on the type of N-species, oxygen content, density of defects and electrical conductivity of the graphene-type materials, a new series of experiments were conducted in electrolytes consisting of mixtures of 0.1 M (NH_4_)_2_SO_4_ + 5 mM HNO_3_, 0.1 M (NH_4_)_2_SO_4_ + 1 M HNO_3_ and 0.1 M H_2_SO_4_ + 1 M HNO_3_. As illustrated by the C 1s and N 1s spectra of EGO samples prepared in 50 vol% mixed electrolytes shown in Figure S4, exfoliation in (NH_4_)_2_SO_4_—based electrolytes favors the formation of amine species with respect to NO_2_. The presence of amines and nitro groups was also confirmed by TGA-MS, Figure S3, and by FTIR analysis, Figures S5 and S6.

**Figure 3:**
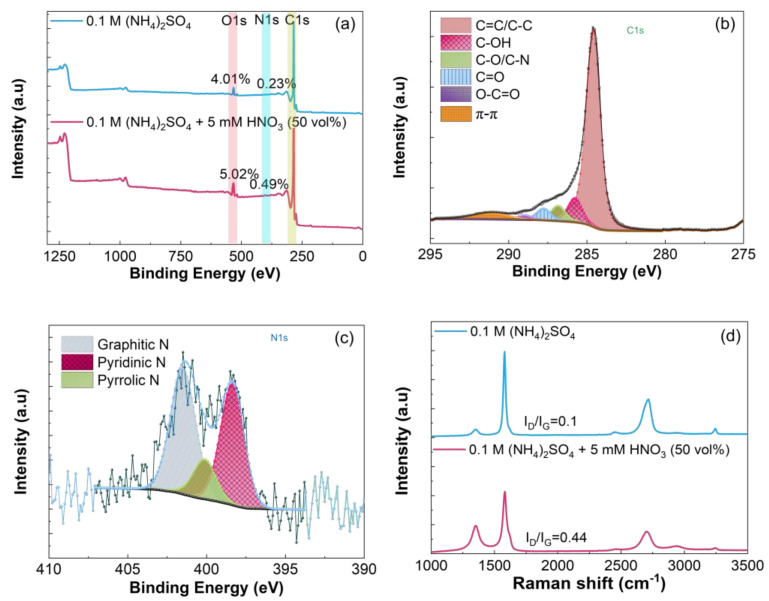
(**a**) XPS survey, (**b**) C1s, (**c**) N1s and (**d**) Raman spectra of REGO obtained using 0.1 M (NH_4_)_2_SO_4_ + 5 mM HNO_3_ (50 vol%) mixed electrolyte, followed by thermal reduction at 900 °C. The XPS survey and Raman spectra of REGO obtained in 0.1 M (NH_4_)_2_SO_4_ were included in Figure (**a**,**d**) for comparison.

**Figure 4:**
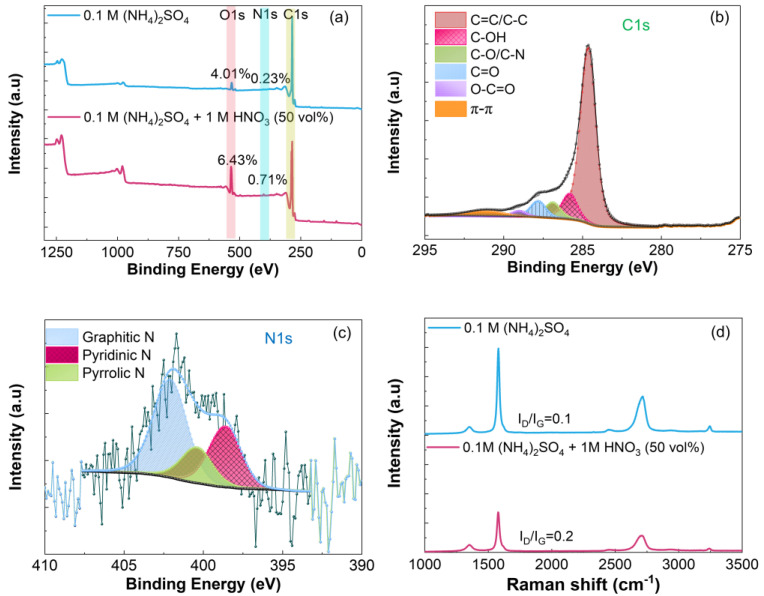
(**a**) XPS survey, (**b**) C1s, (**c**) N1s and (**d**) Raman spectra of REGO obtained using 0.1 M (NH_4_)_2_SO_4_ + 1 M HNO_3_ (50 vol%) mixed electrolyte, followed by thermal reduction at 900 °C. The XPS survey and Raman spectra of REGO obtained in 0.1 M (NH_4_)_2_SO_4_ were included in Figure (**a**,**d**) for comparison.

**Figure 5:**
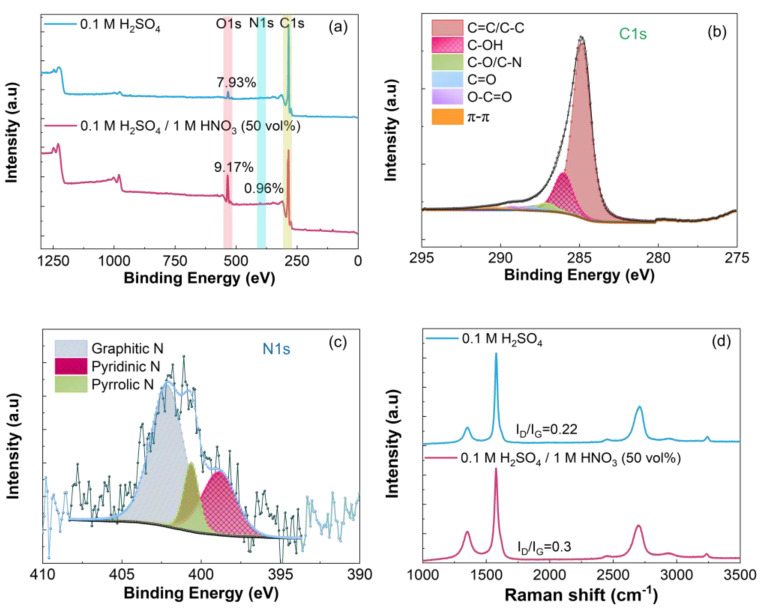
(**a**) XPS survey, (**b**) C1s, (**c**) N1s and (**d**) Raman spectra of REGO obtained using 0.1 M H_2_SO_4_ + 1 M HNO_3_ (50 vol%) mixed electrolyte, followed by thermal reduction at 900 °C. The XPS survey and Raman spectra of REGO obtained in 0.1 M H_2_SO_4_ were included in Figure (**a**,**d**) for comparison.

All samples were thermally reduced at 900 °C. Figure 3, Figure 4 and Figure 5 summarize the XPS and Raman data recorded for samples prepared in 50 vol% mixed electrolytes. The XPS spectra show that the oxygen and nitrogen contents increase when HNO_3_ (50 vol%) is combined with 0.1 M (NH_4_)_2_SO_4_ or 0.1 M H_2_SO_4._ As expected, the increase in the oxygen content is more pronounced with 1 M vs. 5 mM HNO_3_ because of the strong oxidation power of this acid which favors the oxidation of graphite [62], Figure 3a and Figure 5a. The C 1s and N 1s spectra of REGO were deconvoluted the same way as those obtained for REGO prepared in 0.1 M (NH_4_)_2_SO_4_ (Figure 2b and Figure 2d, respectively). The exfoliation in 0.1 M (NH_4_)_2_SO_4_ + 1 M HNO_3_ (50 vol%) increases the fraction of C-O-C/C-N, C=O and O-C=O groups, Figure 4b, compared to 0.1 M (NH_4_)_2_SO_4_ alone, Figure 2b, or to 5 mM HNO_3_ 50 vol%, Figure 3b. Interestingly, mainly C-OH groups are formed when the exfoliation is conducted in 0.1 M H_2_SO_4_ + 1 M HNO_3_ 50 vol%, Figure 5b. The analysis of the N 1s spectra also shows differences in the relative distribution of the nitrogen species with the electrolyte composition, and the largest relative content of pyridinic nitrogen is found when the REGO is prepared from 0.1 M (NH_4_)_2_SO_4_ + 5 mM HNO_3_ 50 vol% Figure 3c. FTIR analysis confirms that the substitutional doping with nitrogen occurs after annealing, Figure S6b.

Despite the increase in oxygen and in nitrogen contents, the I_D_/I_G_ ratios for these REGO samples are relatively low, and the highest one is 0.44 for REGO prepared in 0.1 M (NH_4_)_2_SO_4_ + 5 mM HNO_3_ (50 vol%), Figure 3d, Figure 4d and Figure 5d. This sample’s higher I_D_/I_G_ ratio is counterintuitive, but it will be explained below. Previous works on the electrochemical exfoliation of graphite rods [98] or on the electrochemical oxidation of natural graphite flakes [99] in H_2_SO_4_:HNO_3_ mixtures do not mention the presence of nitrogen species on the products. However, a study on the electrochemical deconsolidation of matrix graphite using HNO_3_ as electrolyte showed an increase of the nitrogen content (from 0.53 to 1.08%) with the 1 M HNO_3_ (from 4 to 68%) [62]. According to the authors, the trend suggests the formation of HNO_3_-graphite intercalation compound. The different observations among experimental works highlights how the process parameters influence the functionalization of graphene-type materials with nitrogen species.

**Figure 6:**
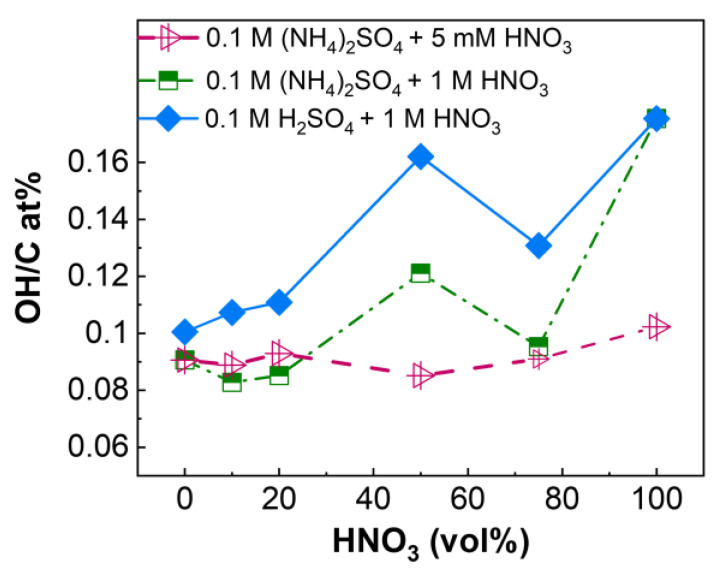
Variation of OH/C at% as a function of the HNO_3_ vol% in the electrolyte.

**Figure 7:**
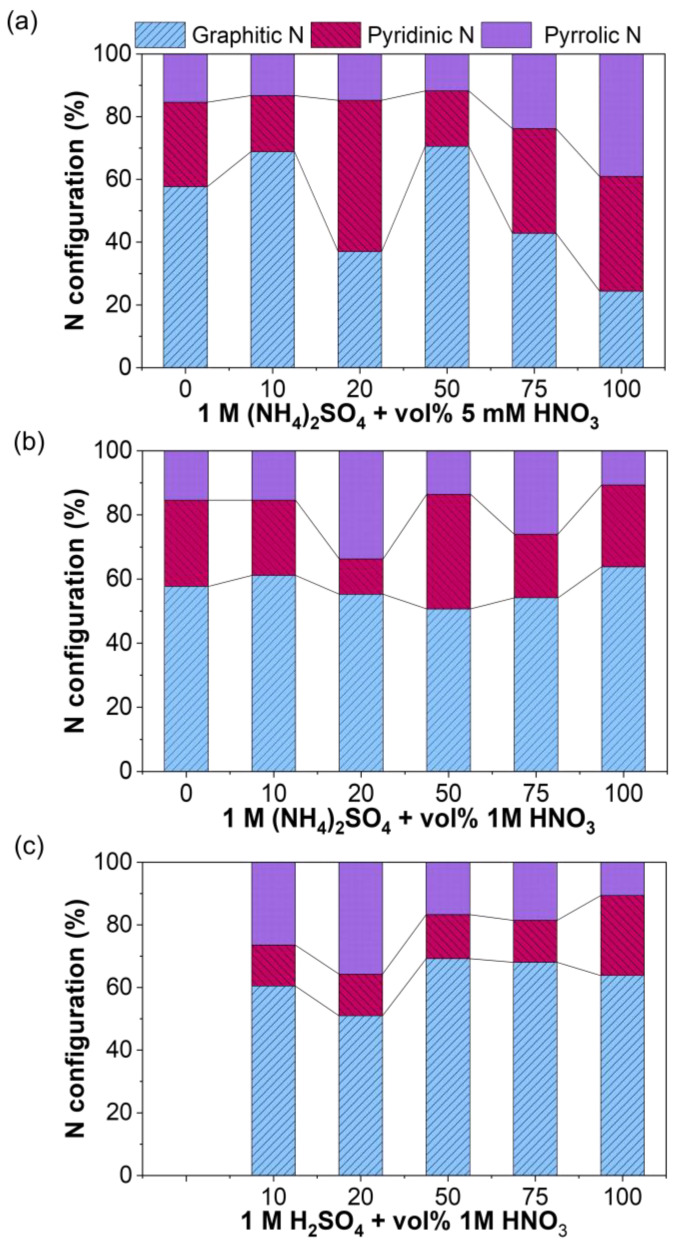
Variation of N at% components in REGO synthesized by electrochemical exfoliation in (**a**) 0.1 M (NH_4_)_2_SO_4_ + 5 mM HNO_3_, (**b**) 0.1 M (NH_4_)_2_SO_4_ + 1 M HNO_3_, (**c**) 0.1 M H_2_SO_4_ + 1 M HNO_3_. All samples were thermally reduced at 900 °C in Ar atmosphere.

The results presented in Figure 3, Figure 4 and Figure 5 anticipate that the relative distribution of the hydroxyl functional groups and nitrogen species in the graphene layers can be highly influenced by the nature of the electrolyte. This is shown in Figure 6 and Figure 7, which present the relative distribution of the C-OH groups and the N-species in all REGO materials prepared using the three series of mixed electrolytes, respectively. According to Figure 6, the fraction of hydroxyl groups is almost constant in the samples obtained using 0.1 M (NH_4_)_2_SO_4_ + 5 mM HNO_3_. However, the OH/C ratio increases from 0.09 to 0.12 and from 0.10 to 016 when the vol% of 1 M HNO_3_ increases up to 50% in the two mixed electrolytes. More importantly, the REGOs produced in the H_2_SO_4_-based mixed electrolyte present the highest OH/C ratio. A larger fraction of pyridinic-N is obtained using 0.1 M (NH_4_)_2_SO_4_ + 5 mM HNO_3_ mixed electrolyte, Figure 7a. Instead, graphitic-N is always the predominant species when 1 M HNO_3_ is used with (NH_4_)_2_SO_4_ and H_2_SO_4_, Figure 7b,c, most likely due to the high temperature (900 ℃) used in the thermal reduction [97]. Nevertheless, using H_2_SO_4_ seems to favor the formation of graphitic-N.

**Figure 8:**
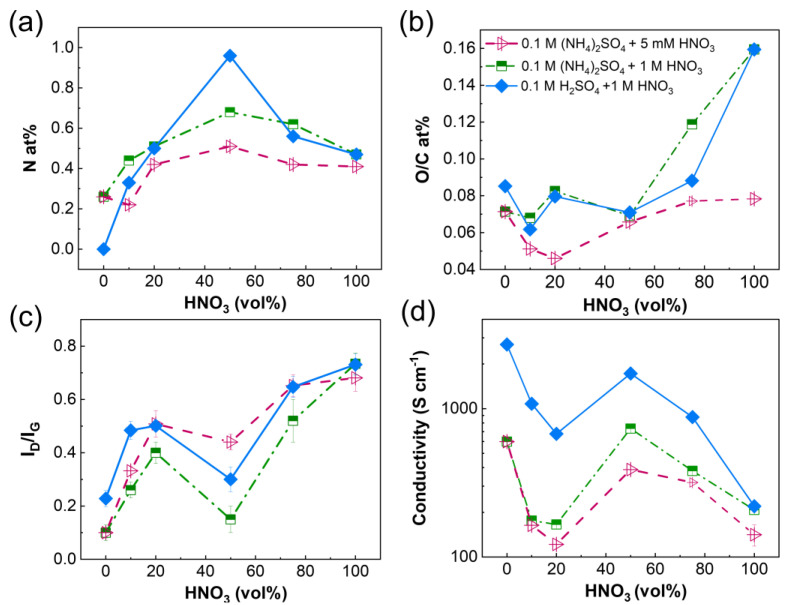
Variation of (**a**) N at%, (**b**) O/C at%, (**c**) I_D_/I_G_ and (**d**) electrical conductivity of the REGO samples as a function of the HNO_3_ vol% in the electrolyte.

Figure 8a–c show the variation of the surface composition and of the I_D_/I_G_ ratio of the REGO samples in the entire range of electrolyte compositions investigated in this work. The N at% increases with the HNO_3_ content until 50 vol%, then decreases for higher vol%, Figure 8a higher doping levels are obtained with 1 M HNO_3_ compared to 5 mM HNO_3_. These two trends put in evidence the benefits of mixing two electrolytes to increase the substitutional doping level. However, the relative concentration of HNO_3_ must be kept below 50 vol% to maximize the N-doping of the REGO flakes and avoid the oxidation of the graphite foil, Figure 8b. The I_D_/I_G_ ratio also increases with HNO_3_ vol%, Figure 8c. However, a singular minimum is found in all cases at 50% vol, consistent with two different situations: anion intercalation, oxidation of graphite and exfoliation at vol% HNO_3_ below 50% and intercalation, oxidation and expansion of graphite oxide for vol% above 50% [62]. Higher I_D_/I_G_ values were obtained for REGO prepared in 0.1 M (NH_4_)_2_SO_4_ + 5 mM HNO_3_ compared to those prepared in 0.1 M (NH_4_)_2_SO_4_ + 1 M HNO_3_. This contrasts with the lower N at% and O/C at% ratio obtained for the former compared to the latter but agrees very well with the higher relative content of pyrrolic and pyridinic nitrogen found in the samples prepared in 0.1 M (NH_4_)_2_SO_4_ + 5 mM HNO_3_, Figure 7a. These species are located at the edges and defects of the graphene sheets and disrupt the carbon sp^2^ domains. Instead, graphitic N does not cause defects [74].

The electrical conductivity of all REGO samples was measured, and the results are reported in Figure 8d. First, the electrical conductivity is the highest for the REGO materials obtained from the 0.1 M H_2_SO_4_ + 1 M HNO_3_, followed by the 0.1 M (NH_4_)_2_SO_4_ + 1 M HNO_3_ series, and then by the 0.1 M (NH_4_)_2_SO_4_ + 5 mM HNO_3_ series. The highest conductivity value (2705 Scm^−1^) was obtained for REGO prepared in 0.1 M H_2_SO_4_. For the doped samples, it varies between 1750 Scm^−1^ and 675 Scm^−1^ for REGO prepared in the 0.1 M H_2_SO_4_ + 50 vol% and 20 vol% 1 M HNO_3_, respectively. The electrical conductivity of REGO obtained in 0.1 M (NH_4_)_2_SO_4_ is 600 Scm^−1^. It decreases to 166 Scm^−1^ for the sample prepared in the mixed electrolyte containing 20 vol% 0.1 M HNO_3_, but it increases to 735 Scm^−1^ for the REGO prepared in 0.1 M (NH_4_)_2_SO_4_ + 50 vol% 1 M HNO_3_. Some of the conductivity values measured in this work are very high compared to those reported previously in the literature for nitrogen-doped reduced graphene oxide obtained by chemical methods, Table S2 [22,48,58,59,100,101,102,103]. Electrical conductivity in the order of 2700 Scm^−1^ was reported for undoped graphene synthesized by RF magnetron deposition sputtering [101], while the electrical conductivity of 3112 Scm^−1^ was reported for graphene oxide obtained by the Hummers method followed by reduction under Joule-Heating process at 2750 K [103]. Instead, the highest value reported for materials synthesized by a chemical method is 800 Scm^−1^ [77]. A visual comparison between the reported literature and our results is done in Figure S7 where the conductivity is plotted against I_D_/I_G_. The figure confirms that graphite exfoliation followed by thermal reduction can produce highly conductive and/or low-defect graphene-based materials.

The variation of the conductivity vs. vol% HNO_3_ in the three series mirrors the variation of I_D_/I_G_ ratio vs. vol% HNO_3_, with a decrease (increase) in the conductivity values corresponding to an increase (decrease) of the I_D_/I_G_ ratio. The higher conductivity vs. lower I_D_/I_G_ ratio relationship also holds for the differences in magnitude found for the series of samples obtained in (NH_4_)_2_SO_4_ mixed with 1 M and 5 mM HNO_3_. However, the REGO materials obtained from 0.1 M H_2_SO_4_ + 1 M HNO_3_ electrolytes have the highest conductivity values and yet intermediate I_D_/I_G_ values compared to the other two series. This suggests that for this series of materials, there is a larger number of sp^2^ domains but smaller dimensions [104]. This hypothesis agrees well with the highest fraction of OH groups found for this series of samples (Figure 6), as the hydroxyl groups are primarily present on the edges of the graphene structure after thermal reduction [105].

**Figure 9:**
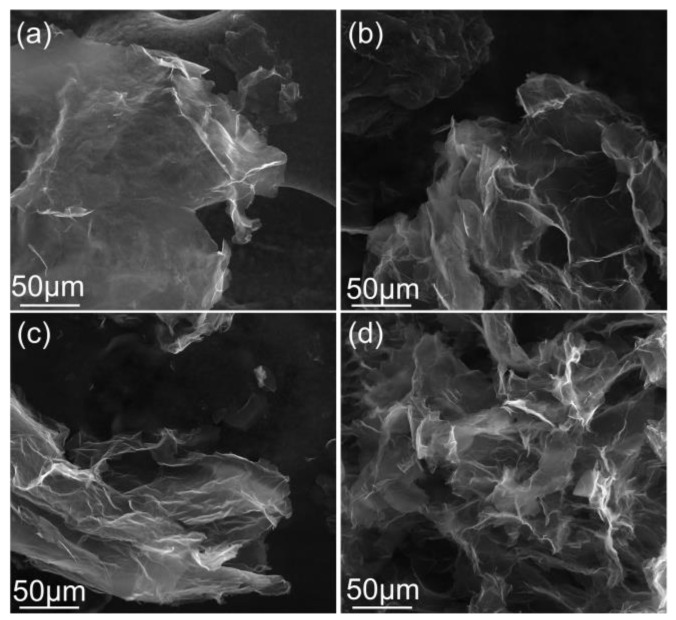
SEM images of REGO layers synthesized with (**a**) 0, (**b**) 10, (**c**) 20, (**d**) 50 vol% of 1 M HNO_3_ mixed with 0.1 M H_2_SO_4_.

The morphology of the REGO sheets obtained from this mixture of electrolytes was investigated by SEM, Figure 9. The electrochemical exfoliation of graphite by H_2_SO_4_ provides large flakes that exceed 200 µm^2^. As the doping level increases in the REGO, the sheets are more wrinkled, but the sheets are still large. The morphology of REGO and of graphite foil (Figure S8) are clearly distinct. Despite the presence of dopants and defects, preserving large sheet sizes could also explain the high conductivity of the REGO materials prepared in this work, especially when using H_2_SO_4_ as the exfoliation electrolyte. Consequently, this method produces large graphene sheets with controllable morphology, useful for several applications such as batteries, fuel cells, supercapacitors and sensors. However, the specific surface area of the materials obtained by this method is typically in the range of 25 m^2^g^−1^ [38]. Finally, analysis of the Raman spectra (fitting of the 2D peak, Figures S9–S11 and I_G_/I_2D_ integrated intensities ratio, Table S3) [69,70,71] and HR-TEM/SAED observations on selected samples (Figures S12 and S13) indicate that the REGO are mostly bilayer materials, except when 100 vol% HNO_3_ is used. In these later conditions, 3–7 layers of graphene-based materials are obtained.

### 3.3. Stability and Purity of REGO Samples after Exposure to Air

The stability and purity of the synthesized REGO were verified by XRD, HR-TEM and XPS analysis of selected samples as prepared and exposed to air for three months. The XRD patterns reported in Figure S14 show a diffraction peak at 2θ = 26⁰ characteristic of a few layers of graphene-based materials, and no additional phases were detected. The d-spacing (interlayer distance) corresponding to the (002) diffraction peak is centered at 3.36 Å (Table S5), as expected [106], and remains almost invariable after 3 months of storage. The (001) interplanar distance was calculated from the SAED patterns and as reported in Table S5 almost no variation is found after storage. However, the presence of large or multiple adjacent diffraction spots in SAED images (Figure S13) indicates the stacking of the graphene layers with time. XPS analysis revealed that the atomic concentrations of nitrogen, oxygen and carbon were almost unchanged after 3 months (Figures S15 and S16, Table S6). No other elements were detected. Thus, the storage of REGOs for 3 months in air does not influence the crystal structure and chemical composition of the synthesized graphenic materials.

## 4. Conclusions

Electrochemical exfoliation of graphite foils in N-containing electrolytes followed by thermal reduction at 900 °C is a powerful method to prepare N-doped REGO few layers materials with a low density of defects (0.1 ≤ I_D_/I_G_ ≤ 0.4) and very high electrical conductivity (121 ≤ σ ≤ 1724 Scm^−1^). Undoped REGO with an electrical conductivity as high as 2705 Scm^−1^ was obtained by electrochemical exfoliation of graphite foil in 0.1 M H_2_SO_4_.

The systematic study on the effect of the electrolyte composition on the properties of the materials prepared in this work showed the benefits of mixing two electrolytes to increase the N-substitutional doping level, keeping a low density of defects at the same time. A more significant fraction of pyridinic-N was obtained using 0.1 M (NH_4_)_2_SO_4_ + 5 mM HNO_3_ mixed electrolytes. Instead, graphitic-N is always the predominant species when electrolytes containing 1 M HNO_3_ are used.

The effect of the nature of the oxygenated functional groups on the electrical conductivity of the REGO is also highlighted. More specifically, the addition to the electrolytes of 1 M HNO_3_ up to 50 vol% leads to samples with a larger fraction of hydroxyl groups after the thermal reduction and higher electrical conductivity. Additionally, the stability of the crystal structure and composition of the materials under 3 months of storage in air was demonstrated by XPS, XRD, HR-TEM and SAED. However, stacking of the graphitic layers occurs over time.

Finally, the exfoliation of graphite foils in N-containing electrolytes leads to graphene oxide materials functionalized with amine and nitro functional groups. A thermal treatment is needed for the substitutional N-doping of the graphene layers.

The synthesis method used in this work produces high-quality (low density of defects), large graphene sheets with tunable composition and electrical conductivity that could be useful for several applications such as batteries, fuel cells, supercapacitors and sensors. The size distribution of the freshly prepared EGO sheets is large, but sonication helps reduce and uniformize their sizes. Moreover, the specific surface area of the materials obtained by this method may be too low for applications in catalysis and electrocatalysis. Filtration and centrifugation of REGO’s suspensions can be used to obtain fractions of materials with selected sizes. Better control of the sheet size during the electrochemical process is highly desirable to reduce the steps involved in the synthesis of the materials.

## Data Availability

The data presented in this study are available on request to the corresponding authors.

## Supplementary Materials

The following supporting information can be downloaded at: https://www.mdpi.com/article/10.3390/nano14010123/s1, Raman spectra of graphite foil and REGO samples prepared by electrochemical exfoliation of graphite foil in 0.1 M (NH_4_)_2_SO_4_ for 1 h, 6 h and 12 h, followed by thermal reduction in Ar atmosphere at 900 °C for 1 h, Figure S1; Analysis of the Raman spectra (integrated intensities I_G_/I_2D_ ratio) of REGO samples prepared in (NH_4_)_2_SO_4_, Table S1; FTIR spectra of EGO and REGOs synthesized by electrochemical exfoliation of graphite foil in 0.1 M (NH_4_)_2_SO_4_ for 1 h, 6 h and 12 h. The reduced samples were obtained after thermal annealing in Ar atmosphere at 900 °C for 1 h, Figure S2; TGA, DTG and MS profiles recorded for EGO obtained by exfoliation of graphite foils in 0.1 M H_2_SO_4_, 0.1 M (NH_4_)_2_SO_4_ and in mixed electrolytes containing HNO_3_, Figure S3; C1s, N1s core level of EGO obtained using 0.1 M (NH_4_)_2_SO_4_ + 5 mM HNO_3_ (50 vol%), 0.1 M (NH_4_)_2_SO_4_ + 1 M HNO_3_ (50 vol%) and 1 M H_2_SO_4_ + 1 M HNO_3_ (50 vol%) mixed electrolytes, Figure S4; FTIR spectra of EGO and REGOs synthesized by electrochemical exfoliation of graphite foil in 0.1 M H_2_SO_4_ and 0.1 M (NH_4_)_2_SO_4_ for 1 h, Figure S5; FTIR spectra of EGO and REGOs synthesized by electrochemical exfoliation of graphite foil in 0.1 M H_2_SO_4_ + 1 M HNO_3_ (50 vol%), 0.1 M (NH_4_)_2_SO_4_ + 1 M HNO_3_ (50 vol%) and 0.1 M (NH_4_)_2_SO_4_ + 5 mM HNO_3_ (50 vol%) for 1 h, Figure S6; Reduced graphene oxide properties reported in this work and in the literature, Table S2; I_D_/I_G_ as a function of electrical conductivity (Scm^−1^) reported in the literature, Figure S7; SEM images of graphite foil, Figure S8; Raman spectra of graphite foil and REGO obtained using 0.1 M (NH_4_)_2_SO_4_ + 5 mM HNO_3_ electrolytes mixtures along with the deconvolution of 2D peak, Figure S9; Raman spectra of graphite foil and REGO using 0.1 M (NH_4_)_2_SO_4_ + 1 M HNO_3_ electrolytes mixtures along with the deconvolution of 2D peak, Figure S10; Raman spectra of graphite foil and REGO using 0.1 M H_2_SO_4_ + 1 M HNO_3_ electrolytes mixtures electrolyte along with the deconvolution of 2D peak, Figure S11; Analysis of the REGO Raman spectra: integrated intensities I_G_/I_2D_ ratio, Table S3; TEM images and respective SAED patterns of REGO after thermal reduction at 900 °C and 3 months of storage in air, Figure S12; D-spacing corresponding to the (100) plane (SAED), Table S4; HR-TEM and SAED of REGO synthesized in 1 M HNO_3_, Figure S13; XRD patterns of REGO synthesized in different electrolytes mixtures (a) after reduction (b) after 3 months of exposure to air, Figure S14; D-spacing (interlayer distance) calculated from the (002) diffraction peak (X-ray diffraction), Table S5; XPS survey spectra of REGO obtained using (a) 0.1 M (NH_4_)_2_SO_4_, (b) 0.1 M (NH_4_)_2_SO_4_ + 5 mM HNO_3_ (50 vol%), (c) 0.1 M (NH_4_)_2_SO_4_ + 1 M HNO_3_ (50 vol%), (d) 0.1 M H_2_SO_4_ + 1 M HNO_3_ (50 vol%) mixed electrolyte after 3 months of exposure to air, Figure S15; N 1s core level spectra of REGO obtained using (a) 0.1 M (NH_4_)_2_SO_4_, (b) 0.1 M (NH_4_)_2_SO_4_ + 5 mM HNO_3_ (50 vol%), (c) 0.1 M (NH_4_)_2_SO_4_ + 1 M HNO_3_ (50 vol%), (d) 0.1 M H_2_SO_4_ + 1 M HNO_3_ (50 vol%) mixed electrolyte, after 3 months of exposure to air, Figure S16; Surface atomic composition of REGO, after 3 months storage in air, Table S6. References [107,108,109,110,111,112,113,114,115,116] are cited in the supplementary materials.
