# Correction: Kammoun et al. Nitrogen-Doped Graphene Materials with High Electrical Conductivity Produced by Electrochemical Exfoliation of Graphite Foil. *Nanomaterials* 2024, *14*, 123

**DOI:** 10.3390/nano16050318

**Published:** 2026-03-03

**Authors:** Hela Kammoun, Benjamin D. Ossonon, Ana C. Tavares

**Affiliations:** Centre Énergie Matériaux Télécommunications, Institut National de la Recherche Scientifique, 1650 Boulevard Lionel-Boulet, Varennes, QC J3X 1P7, Canadabenjamin.ossonon@inrs.ca (B.D.O.)

**Error in Figure**

In the original publication [1], Figure 2a,b are swapped. The x-axis scales in the middle panels of Figure 2d,f are different (from 0 to 15 h) from those of the bottom and top panels (from 0 to 13 h). The authors requested an update to the Figures.

The correct Figure 2 and Figure caption appear below.

**Text Correction**

The sentence in Section 3.1, paragraph 6, “Secondary amines are formed by the reduction of primary amines, according to Equation (6)”, was changed to “Secondary amines are converted to primary amines, according to Equation (6)”.

The authors state that the scientific conclusions are unaffected. These corrections were approved by the Academic Editor. The original publication has also been updated.

## Figures and Tables

**Figure 2:**
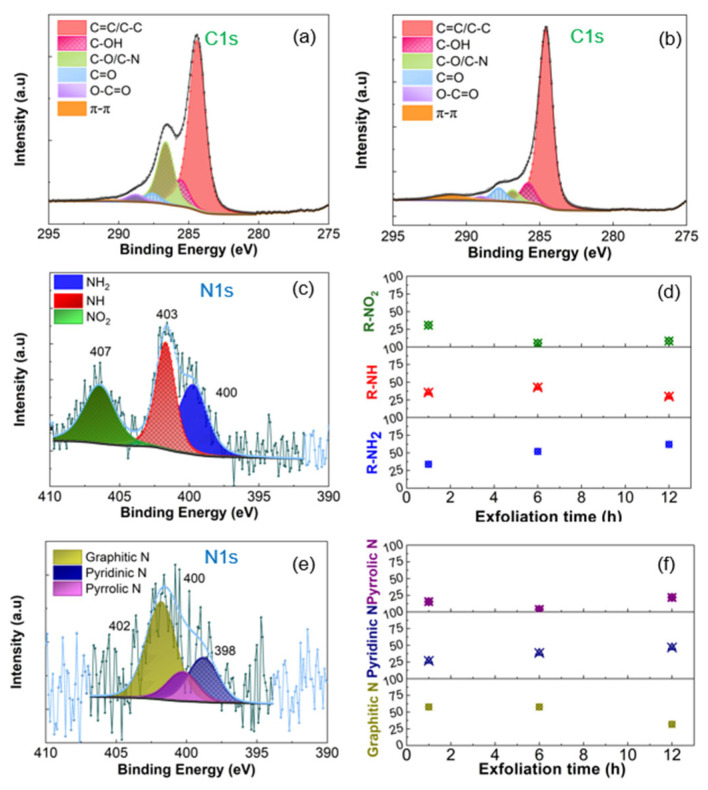
C 1s and N 1s core level spectra of (**a**,**c**) EGO and (**b**,**e**) REGO. Effect of exfoliation time on the type of N-species (**d**) before and (**f**) after thermal reduction at 900 °C for 1 h. The electrochemical exfoliation of the graphite foil was performed in 0.1 M (NH_4_)_2_SO_4_.
