# Supplementary material for: Nitrogen-Doped Graphene Materials with High Electrical Conductivity Produced by Electrochemical Exfoliation of Graphite Foil
Source: Nanomaterials (Basel). 2024 Jan 4;14(1):123. doi: 10.3390/nano14010123 (PMC10780345; doi:10.3390/nano14010123)
Supplement: Supplementary file 1 [file nanomaterials-14-00123-s001.zip › nanomaterials-2769023-supplementary.pdf]

Supporting information

Nitrogen-Doped Graphene Materials with High Electrical Conductivity Produced by Electrochemical Exfoliation of Graphite Foil

Hela Kammoun, Benjamin D. Ossonon † and Ana C. Tavares *

Centre Énergie Matériaux Télécommunications, Institut National de la Recherche Scientifique,
1650 Boulevard Lionel-Boulet, Varennes, QC J3X 1P7, Canada; hela.kammoun@inrs.ca (H.K.);
benjamin.ossonon@inrs.ca (B.D.O.)
* Correspondence: ana.tavares@inrs.ca; Tel.: +1-(514)-2286947
† Current address: UFR des Sciences Biologiques, Mathématique–Physique et chimie, Université Peleforo Gon Coulibaly (UPGC), BP1328 Korhogo, Côte d'Ivoire.

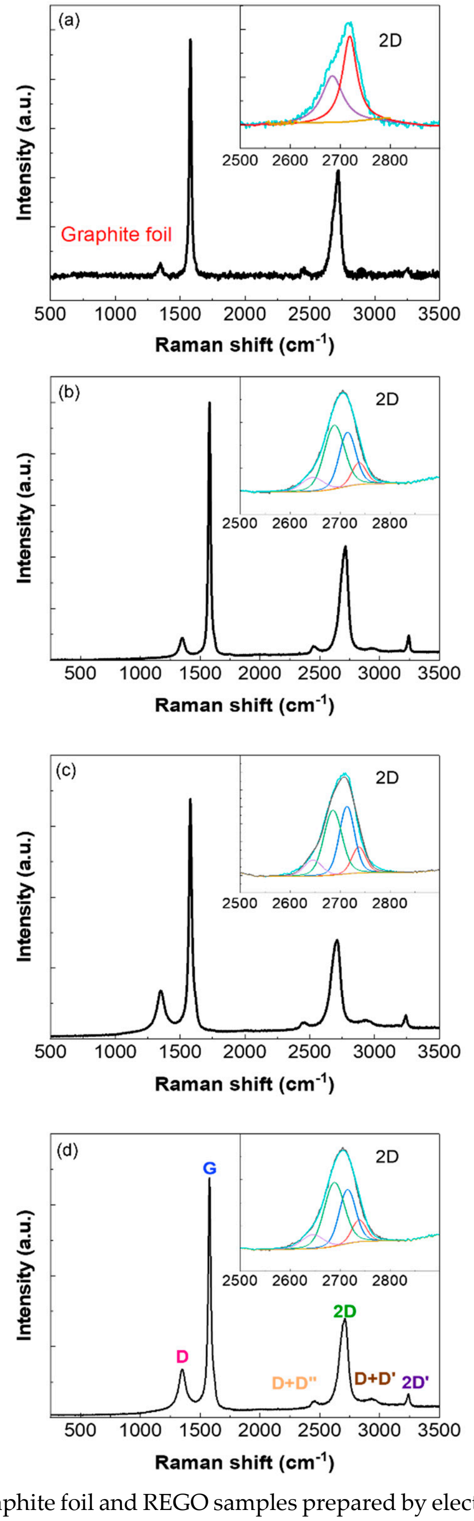

Figure S1. Raman spectra of (a) graphite foil and REGO samples prepared by electrochemical exfoliation of graphite foil in 0.1 M $(NH_4)_2SO_4$ for (b) 1h, (c) 6h and (d) 12h, followed by thermal reduction in Ar atmosphere at 900 °C for 1h. The insets show the deconvolution of the 2D peak.

As expected, the Raman spectrum of the graphite foil shows a low intensity D peak, a high intensity G peak and a sharp 2D peak. Compared to the graphite foil, the Raman spectra of the EGO materials show a more intense D peak and a broader 2D peak. This is related to the breathing mode of the k-points phonon of A1g symmetry associated to the intervalley phonon and scattering defects [1]. The 2D peak was deconvoluted using four Lorentzian components, indicating that a bilayer graphene material was obtained by electrochemical exfoliation [2-5].

The ratio of G and 2D integrated intensities varies between 0.34 and 0.41, Table S1, which indicates that the REGO synthesized in $(NH_4)_2SO_4$ is mostly mono/bilayer [6,7].

Table S1. Analysis of the Raman spectra of REGO prepared in $(NH_4)_2SO_4$: integrated intensities I_G/I_{2D} ratio.

$(NH_4)_2SO_4$	I_G/I_{2D}
**1h**	0.34
**6h**	0.38
**12h**	0.41

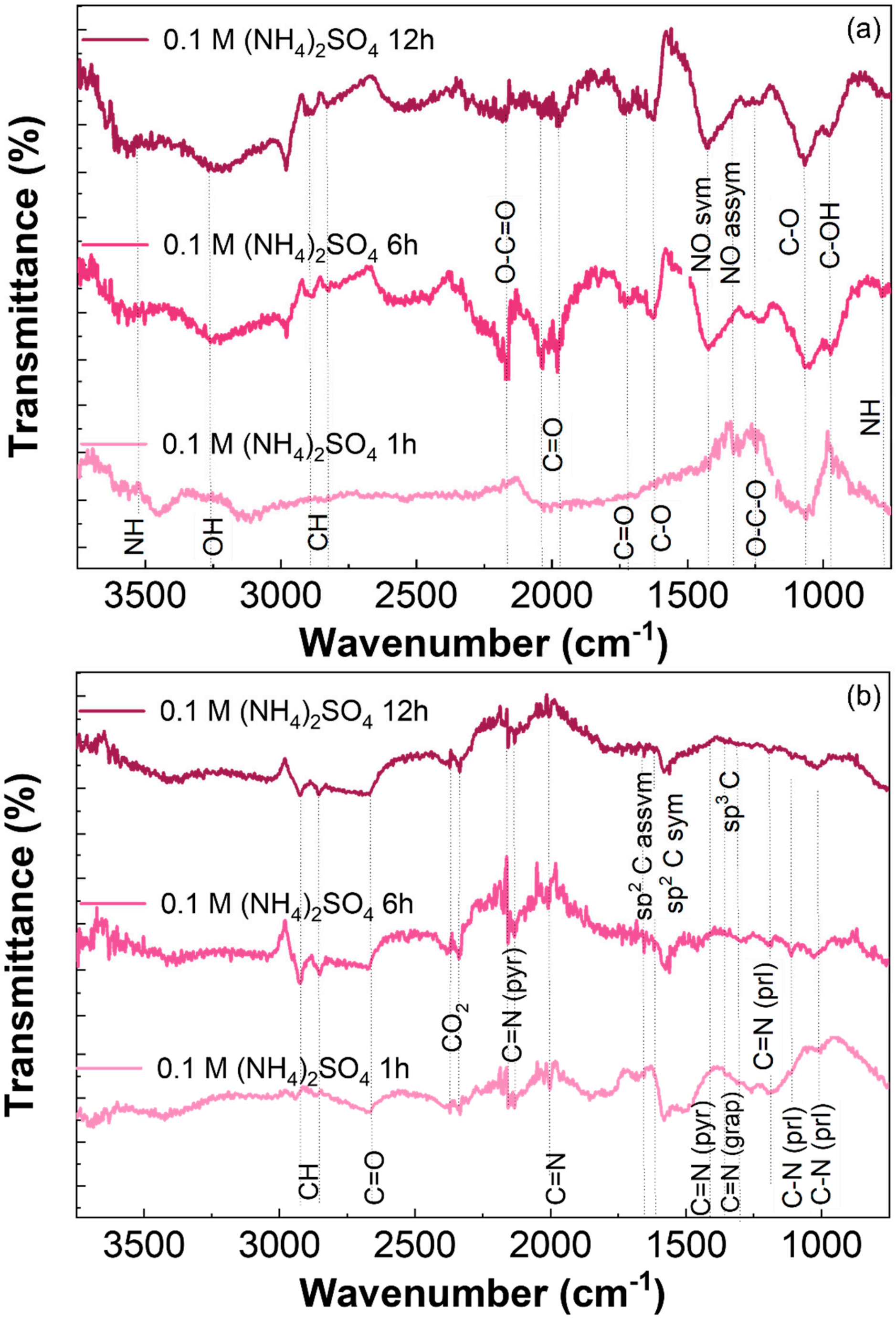

Figure S2. FTIR spectra of (a) EGO and (b) REGOs synthesized by electrochemical exfoliation of graphite foil in 0.1 M $(NH_4)_2SO_4$ for 1 h, 6 h and 12 h. The reduced samples were obtained after thermal annealing in Ar atmosphere at 900 °C for 1 h. Legend: pyr : pyridinic, prl: pyrollic, grap: graphitic.

The FTIR spectra of EGO in Figure S2.a show a broad peak at 3250 cm^{-1} attributed to the stretching mode of O-H bond, consistent with the presence of hydroxyl groups. The bands observed at 1720, 2000 and 2168 cm^{-1} are assigned to the carboxyl group (C=O) [8,9]. The peak at 1236 cm^{-1} denotes O-C-O stretching, the peak at 1056 cm^{-1} is assigned to the stretching mode of the C-O bond [10], and the peak at 935 cm^{-1} corresponds to the vibrational mode of the C-OH group. The bands observed at 780 and 3520 cm^{-1} reveal the presence of amines on the graphene oxide's surface [11,12]. The bands observed at 1330 cm^{-1} and 1416 cm^{-1} are related to nitro compounds [13,14]. Therefore, the XPS and FTIR analysis reveal the formation of primary and secondary amines, and nitro groups during the exfoliation of graphite foil in $(NH_4)_2SO_4$.

The FTIR spectra of the reduced samples are presented in Figure S2.b. The characteristic stretching vibrations of sp^3 and sp^2 carbon appear at 1312 and 1600-1650 cm^{-1}, respectively. The presence of pyridinic nitrogen is confirmed by the two bands at around 1580 and 2157 cm^{-1} [15], pyrrolic nitrogen by the bands at ca. 1005 and 1107 cm^{-1} [15,16], and graphitic nitrogen by two weak bands at around 1360-1400cm^{-1} [15].

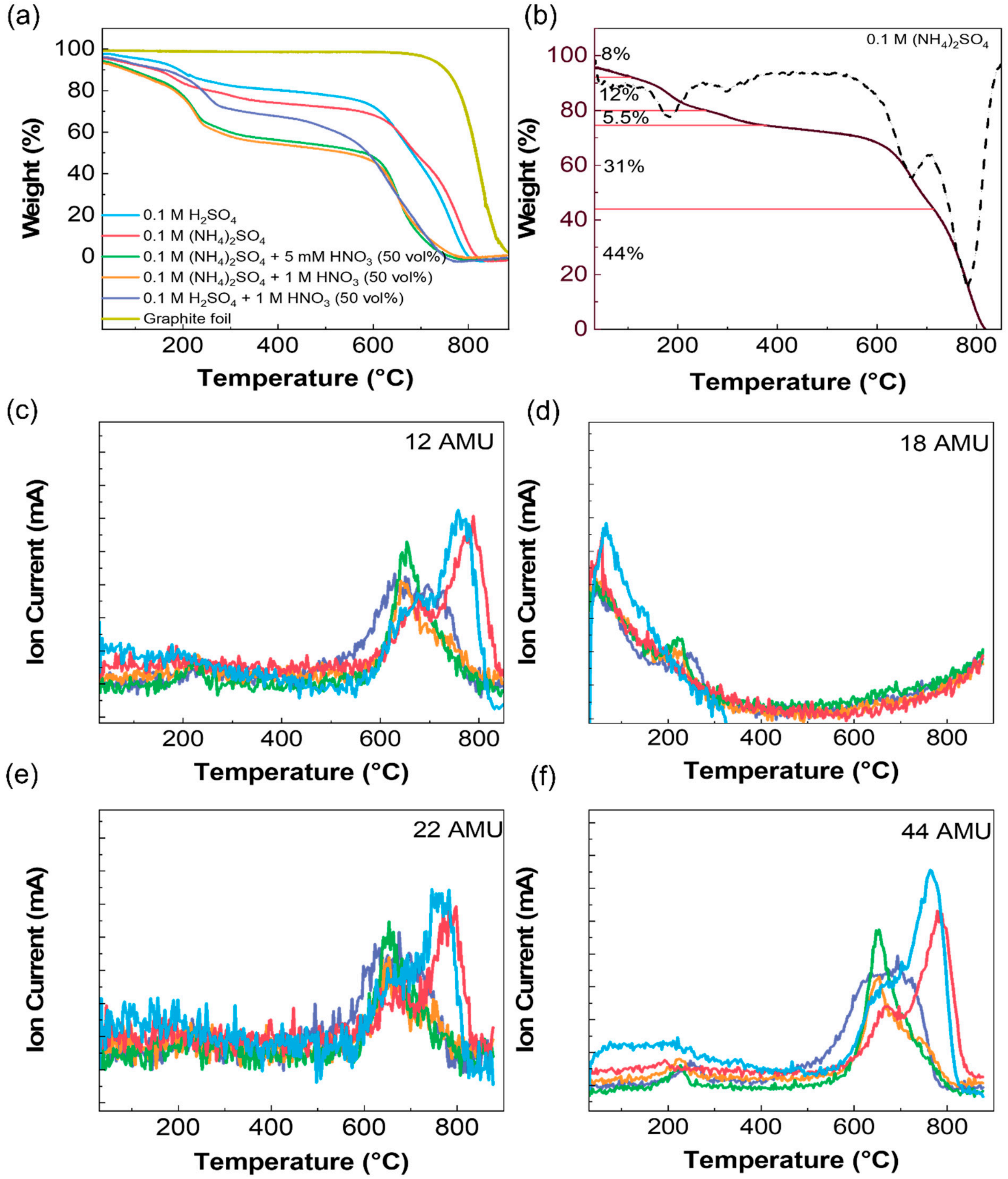

Figure S3. (a) TGA, (b) DTG and (c) to (e) MS profiles recorded for EGO obtained by exfoliation of graphite foils in 0.1 M H_2SO_4, 0.1 M $(NH_4)_2SO_4$ and in mixed electrolytes containing HNO_3. The *m*/*z* values in figures (c) to (e) are 17, 22, and 44 AMU, respectively. The TGA profile of the graphite foil is included in Figure a for comparison.

The thermal analysis of the EGOs shows major losses at 122-227 °C, 273-370 °C, 504-708 °C and 708-850 °C, Figure S3.a, whereas the thermal analysis of graphite shows only one process after 700 °C. The thermal processes of the EGOs are respectively attributed to the loss of adsorbed and intercalated water and primary amines [17]; the decomposition of carboxyl, epoxy or nitro groups [17,18]; decomposition of sp^3 carbon bonded to oxygenated functional groups; and to graphitic carbon [19,20]. The high temperatures of the last two thermal losses confirm that the EGOs prepared in this work are large flakes, low defects and few layers graphene materials [21]. Mass spectrometry corroborated the nature of the chemical moieties released during the TGA experiments. Mass spectra of representative fragments are presented in Figure S3: *m*/*z* = 17 for NH_3^+, *m*/*z* = 22 for CO_2^{2+}, and *m*/*z* = 44 for CO_2^+.

The weight losses in the 273 to 370°C interval for samples prepared in $(NH_4)_2SO_4$ and in the mixed electrolytes are larger and shifted to higher temperature compared to EGO obtained by electrochemical exfoliation in H_2SO_4. The presence of primary and secondary amine groups likely leads to the formation of hydrogen bonds with the oxygenated functional groups of EGO and stabilizes the materials. For EGO obtained in $(NH_4)_2SO_4$ the thermal event associated to the decomposition of graphitic carbon is also shifted to a higher temperature in agreement with the higher quality (lower density of defects) of this material. Instead, this thermal process is significantly attenuated for the samples obtained in the mixed electrolytes, and the decomposition of sp^3 carbon bonded to oxygenated functional groups is clearly shifted to lower temperature. Accordingly, the mass losses for the samples prepared in the electrolyte mixtures with HNO_3 50 vol % are higher than those prepared in H_2SO_4 or $(NH_4)_2SO_4$ alone. Among these three samples, the highest weight losses are found for samples prepared in $(NH_4)_2SO_4$ + HNO_3.

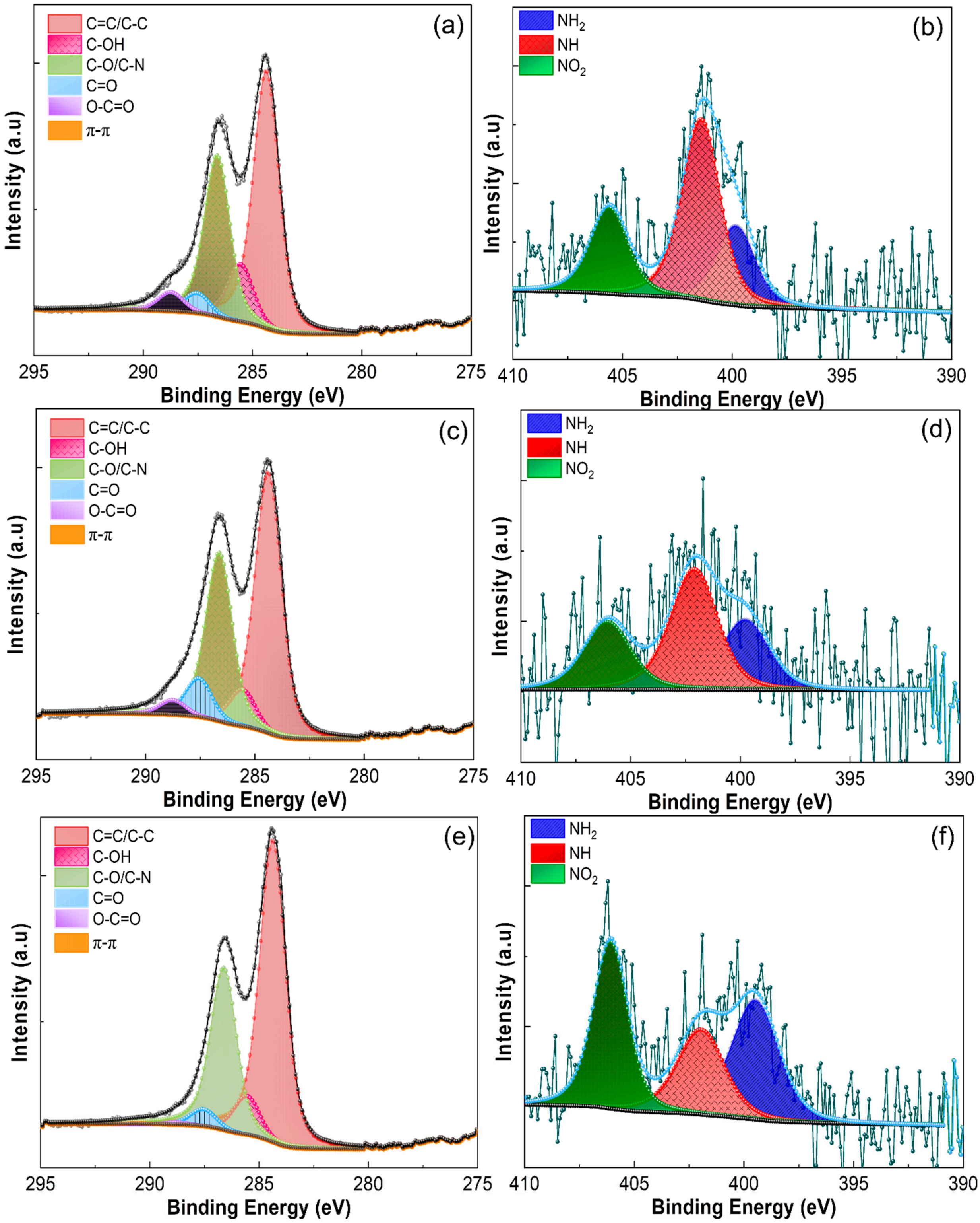

Figure S4. (a) C 1s and (b) N 1s core level spectra of EGO obtained in 0.1 M $(NH_4)_2SO_4$ + 5 mM HNO_3 (50 vol%) mixed electrolyte; (c) C 1s and (d) N 1s core level spectra of EGO obtained using 0.1 M $(NH_4)_2SO_4$ + 1 M HNO_3 (50 vol%) mixed electrolyte; (e) C 1s and (f) N 1s core level spectra of EGO obtained using 0.1 M H_2SO_4 + 1 M HNO_3 (50 vol%) mixed electrolyte.

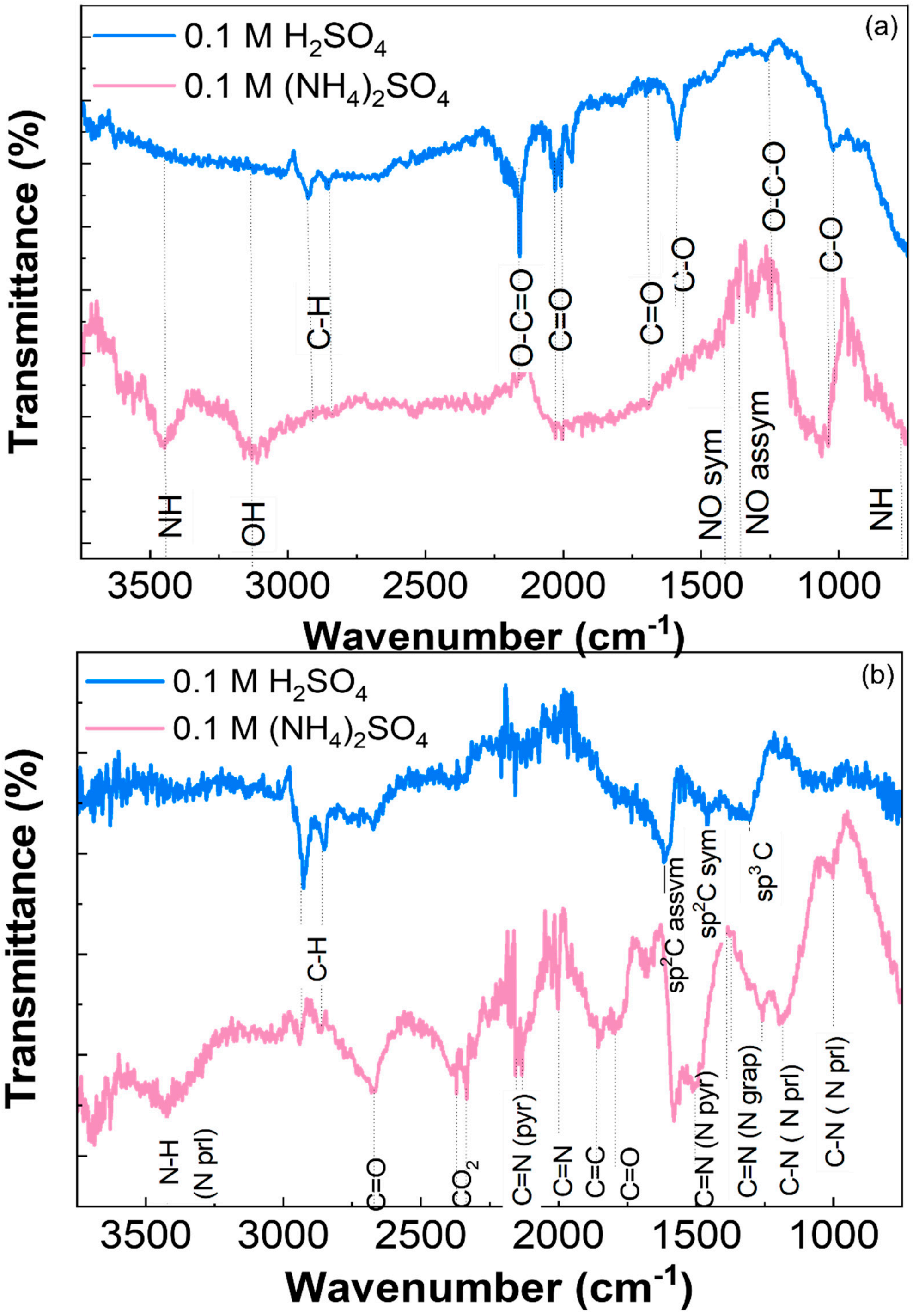

Figure S5. FTIR spectra of (a) EGO and (b) REGOs synthesized by electrochemical exfoliation of graphite foil in 0.1 M H_2SO_4 and 0.1 M $(NH_4)_2SO_4$ for 1h. The reduced samples were obtained after thermal annealing in Ar atmosphere at 900 °C for 1h. Legend: pyr : pyridinic, prl: pyrollic, grap: graphitic.

The FTIR spectra of EGO in Figure S5.a show bands at 1780, 2000 and 2168 cm^{-1} assigned to the carboxyl group (C=O). The peak at 1236 cm^{-1} denotes C-O-C stretching, and the peak at 1056 cm^{-1} corresponds to the vibrational mode of the C-O group. The transmission peaks at 2848 and 2922 cm^{-1} are attributed to the aromatic rings. In the spectrum of EGO synthesized by exfoliation in $(NH_4)_2SO_4$, the bands observed at 780 and 3520 cm^{-1} reveal the presence of amines, whereas those at 1330 and 1416 cm^{-1} correspond to the presence of nitro compounds.

The FTIR spectra of the samples after reduction, Figure S5.b, show the characteristic stretching vibrations of sp^3 carbon at 1310 cm^{-1} and sp^2 carbon at 1470 and 1610 cm^{-1}. The bands related to pyridinic nitrogen (at ca. 1510 and 2139 cm^{-1}), pyrrolic nitrogen (at ca. 1000 and 1200-1266 cm^{-1}), and graphitic nitrogen (weak peaks around 1375-1398 cm^{-1}) are also present in the spectra.

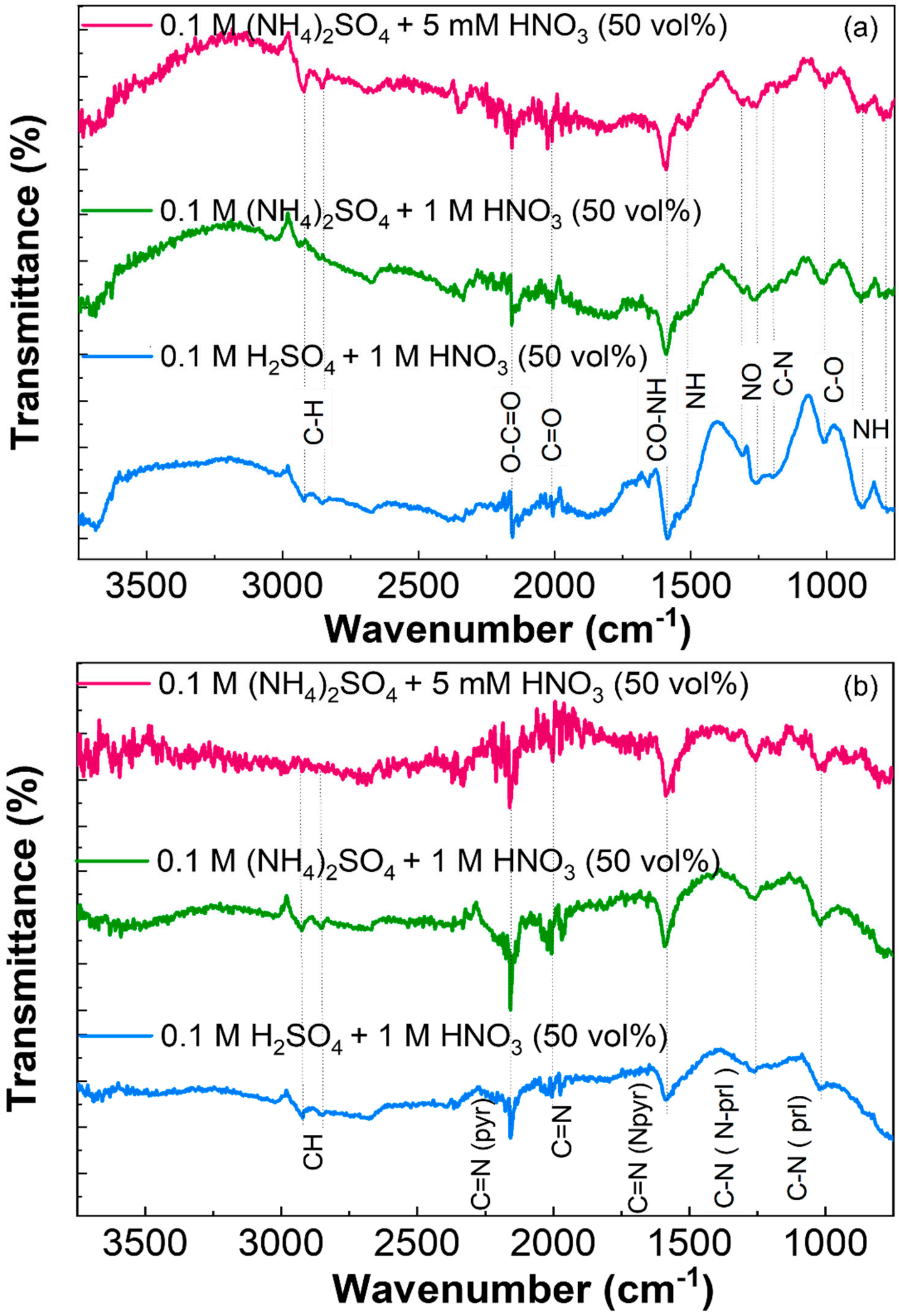

Figure S6. FTIR spectra of (a) EGO and (b) REGOs synthesized by electrochemical exfoliation of graphite foil in 0.1 M H_2SO_4 + 1 M HNO_3 (50 vol%), 0.1 M $(NH_4)_2SO_4$ + 1 M HNO_3 (50 vol%) and 0.1 M $(NH_4)_2SO_4$ + 5 mM HNO_3 (50 vol%) for 1h. The reduced samples were obtained after thermal annealing in Ar atmosphere at 900°C for 1h. Legend: pyr : pyridinic, prl: pyrollic.

Figure S6 depicts the FTIR spectra of EGO obtained in various electrolyte mixtures. The spectra in Figure S6.a show the peaks previously observed with the EGOs prepared by exfoliation in $(NH_4)_2SO_4$ and H_2SO_4 (see Figures 3.a and 5.a). However, the intensity of the peaks associated with nitrogen-containing groups such as NH stretching vibrations at 767-788 cm^{-1} and 1589 cm^{-1}, CN band stretch at 1190 cm^{-1}, nitro groups at 1308 $cm^{-1.}$ is higher than in the spectra of the EGOs exfoliated in the presence of HNO_3 50 vol %.

After thermal reduction, the FTIR spectra (Figure S6.b) present features similar to those of REGO synthesized with H_2SO_4 or $(NH_4)_2SO_4$ ((Figure S5.b). However, the spectra of REGO synthesized in electrolyte mixtures with HNO_3 at 50 vol% (Figure S6.b) shows stronger peaks for pyridinic nitrogen (stretching mode of C=N at 1589 and 2159 cm^{-1}) and pyrrolic nitrogen (stretching mode of C-N at 1017 and 1270 cm^{-1}).

Table S2: Reduced graphene oxide properties reported in this work and the literature.

Synthesis method and conditions	I_D/I_G	O at%	N at%	Electrical conductivity (Scm^{-1})	Ref.
**Electrochemical exfoliation of graphite foil in 0.1 M H_2SO_4; thermal reduction at 900°C under Ar, 1h.**	0.22	7.93%	0	2705	this work
**Electrochemical exfoliation of graphite foil in 0.1 M H_2SO_4 +1 M HNO_3 (50 vol%); thermal reduction at 900°C under Ar,1h.**	0.30	9.17%	0.82	1750	this work
**Electrochemical exfoliation of graphite foil in 0.1 M $(NH_4)_2SO_4$ / 1 M HNO_3 (50 vol%); thermal reduction at 900°C under Ar,1h.**	0.44	5.02%	0.49	733	this work
**Multilayer graphene deposited by RF magnetron deposition sputtering at 200°C**	0.14	n/a	n/a	2700	[22]
**Modified Hummer's method + stirring at 100°C with 0.001 wt.% NH_4OH; thermal reduction at 800°C under N_2, 30 min.**	1.87	9	3	56.2	[23]
**Modified Hummer's method NH_4OH; thermal reduction at 800°C under NH_3 (g), 1h.**	0.89	n/a	0.48	316	[24]
**Modified Hummer's method + reduction in 2 ml of hydrazine at 85°C, 24h; thermal reduction at 800°C under NH_3 (g), 1h.**	0.97	n/a	n/a	52	[24]
**Hydrothermal reaction of $(NH_4)_2CO_3$ /GO (1:100) at 130°C; drying under vacuum at 60°C**	1.28	12.8	10.01	800	[25]
**Alternating current electrochemical exfoliation in TBA·HSO_4**	0.16	21.2	n/a	640	[26]
**Microwave plasma-enhanced chemical vapour deposition**	0.5	32	15	15.32	[27]
**Reduction GO colloids by hydrazine hydrate heating 100°C under a water-cooled condenser 24h**	0.87	2.8	0.06	24	[28]
**Modified Hummer's method reduction of GO film at 773K in Ar + Joule Heating process at 2750 K**	0.02	0.01	n/a	3112	[29]

TBA: tetrabutylammonium

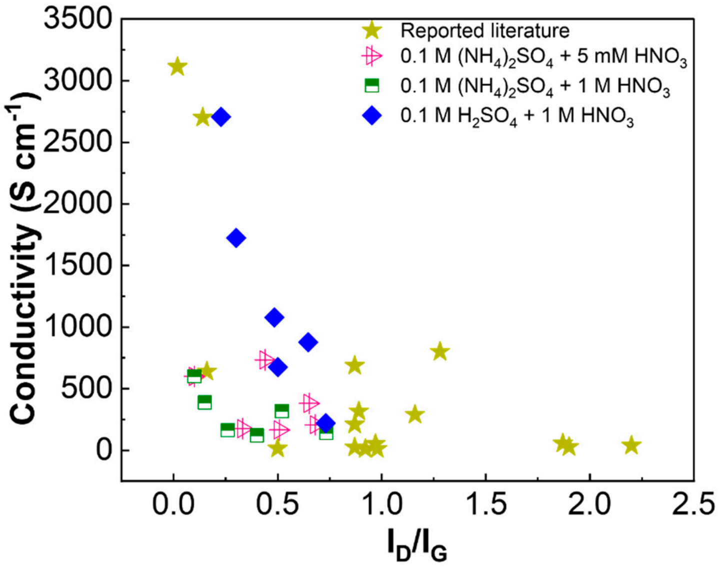

Figure S7. Electrical conductivity as a function of I_D/I_G for reduced graphene materials reported in the literature [22-34] and in this work.

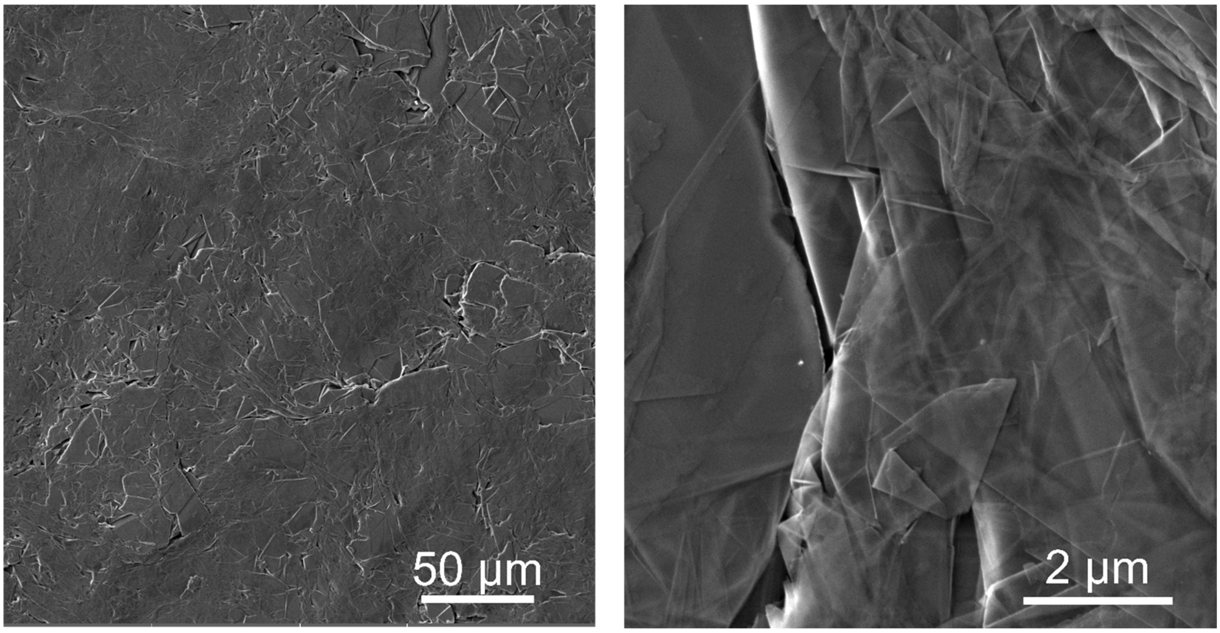

Figure S8. SEM images of graphite foil.

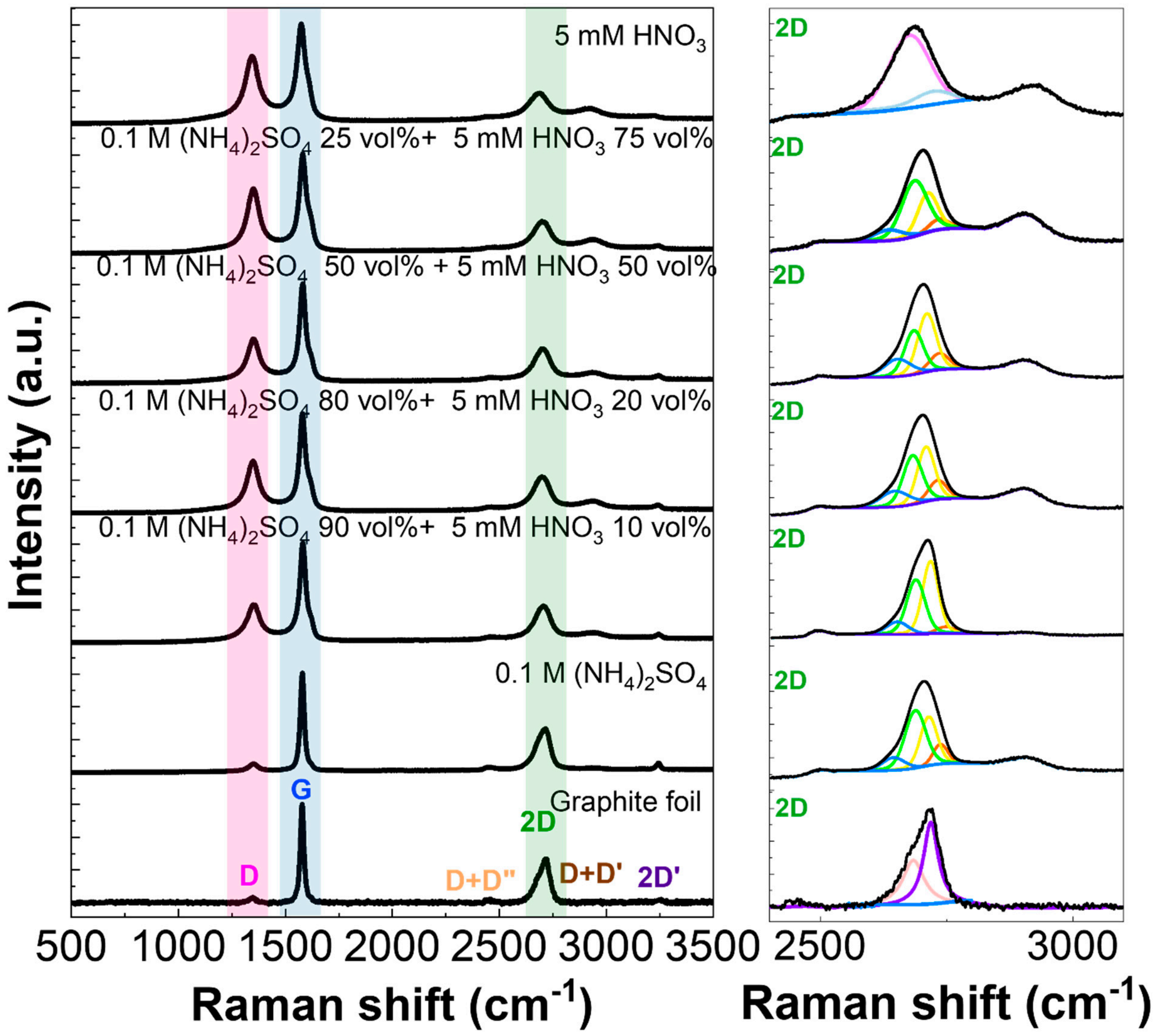

Figure S9. Raman spectra of REGO obtained using 0.1 M $(NH_4)_2SO_4$ + 5 mM HNO_3 electrolytes mixtures along with the deconvolution of 2D peak.

The 2D band is deconvoluted into four components when the graphite is exfoliated in $(NH_4)_2SO_4$ or in the mixed $(NH_4)_2SO_4$ + HNO_3 electrolytes The shape and the deconvolution into four components are characteristic of graphene-based materials mainly composed of two layers. The deconvolution into 2 components in the case of REGO obtained by exfoliation of graphite foil in 100 % HNO_3 indicates a higher number of layers [6].

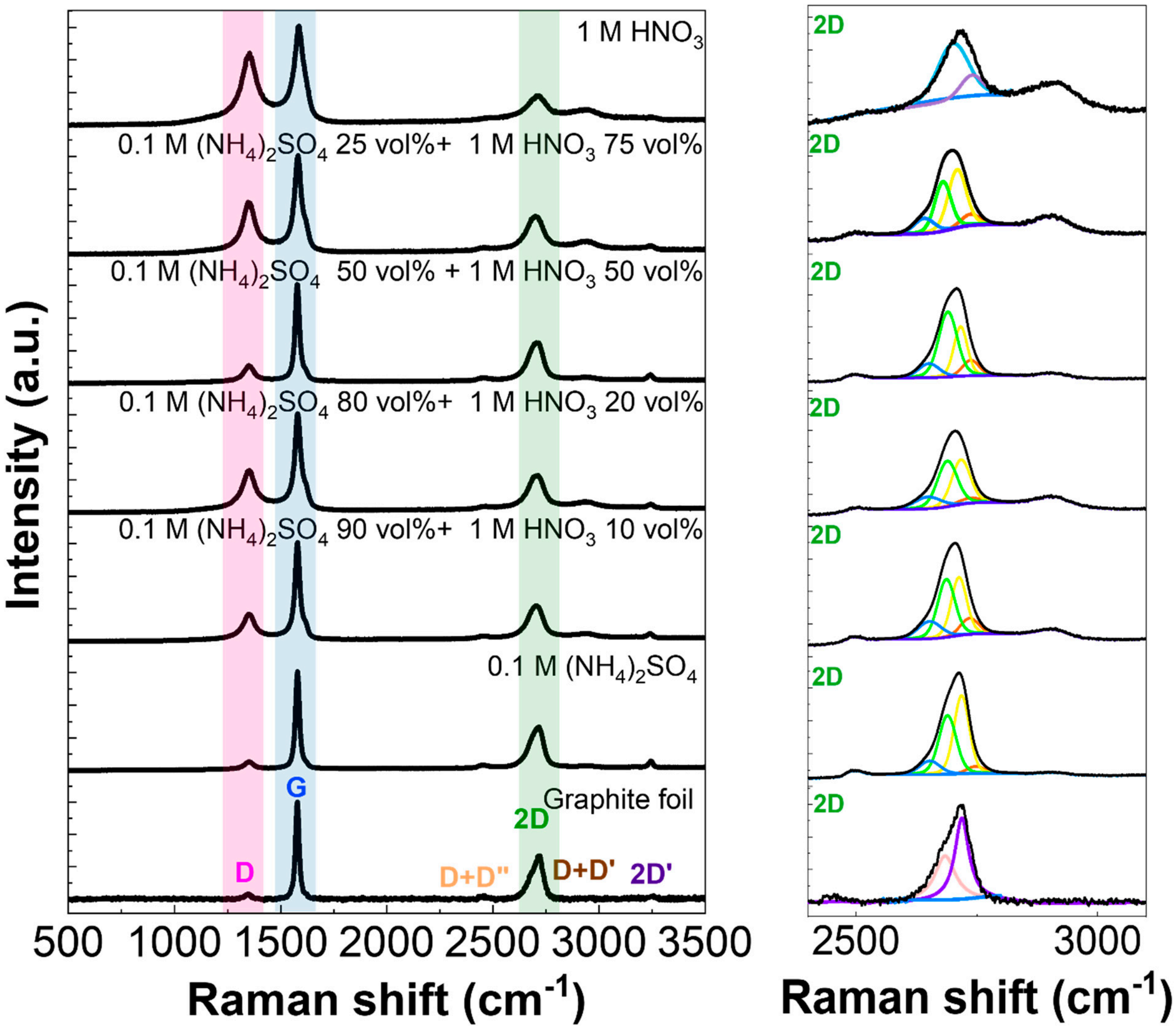

Figure S10. Raman spectra of REGO using 0.1 M $(NH_4)_2SO_4$ + 1 M HNO_3 electrolytes mixtures along with the deconvolution of the 2D peak.

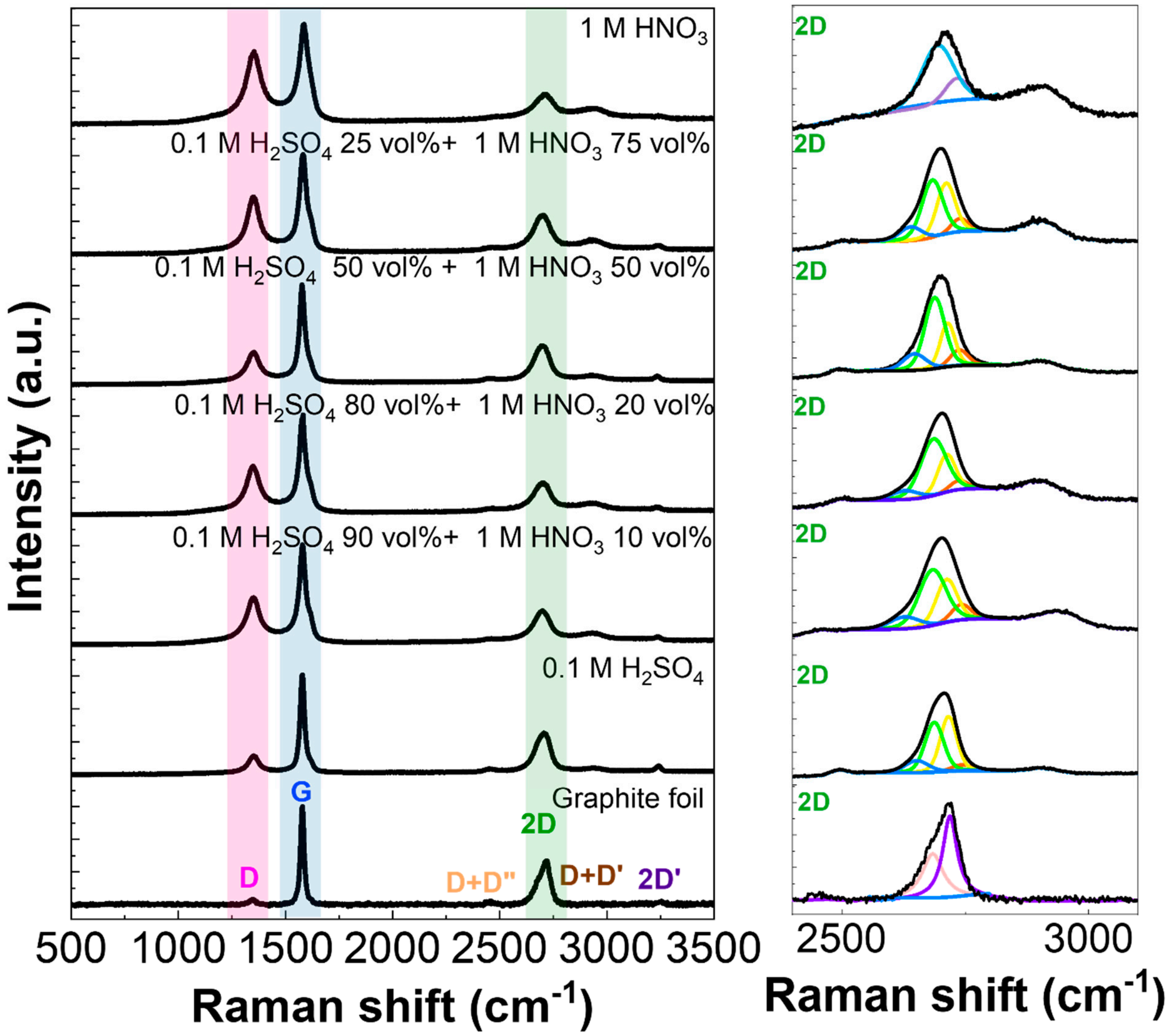

Figure S11. Raman spectra of REGO using 0.1 M H_2SO_4 + 1 M HNO_3 electrolytes mixtures electrolyte along with the deconvolution of the 2D peak.

Table S3. Analysis of the REGO Raman spectra: integrated intensities I_G/I_{2D} ratio

HNO_3 (vol%)	0.1 M $(NH_4)_2SO_4$ + 5 mM HNO_3	0.1 M $(NH_4)_2SO_4$ + 1 M HNO_3	0.1 M H_2SO_4 + 1 M HNO_3
**0**	0.34	0.34	0.32
**10**	0.38	0.37	0.36
**20**	0.39	0.41	0.37
**50**	0.31	0.32	0.36
**75**	0.41	0.39	0.41
**100**	0.63	0.69	0.69

The ratio of G and 2D integrated intensities varies between 0.3 and 0.45 indicates that the REGO synthesized in $(NH_4)_2SO_4$, H_2SO_4 or their mixtures with HNO_3 is mostly mono/bilayer [6,7]. However, the I_G/I_{2D} values above 0.6 confirms the presence of more than two layers when the material is obtained by exfoliation of graphite foil in 100% HNO_3 [6,7].

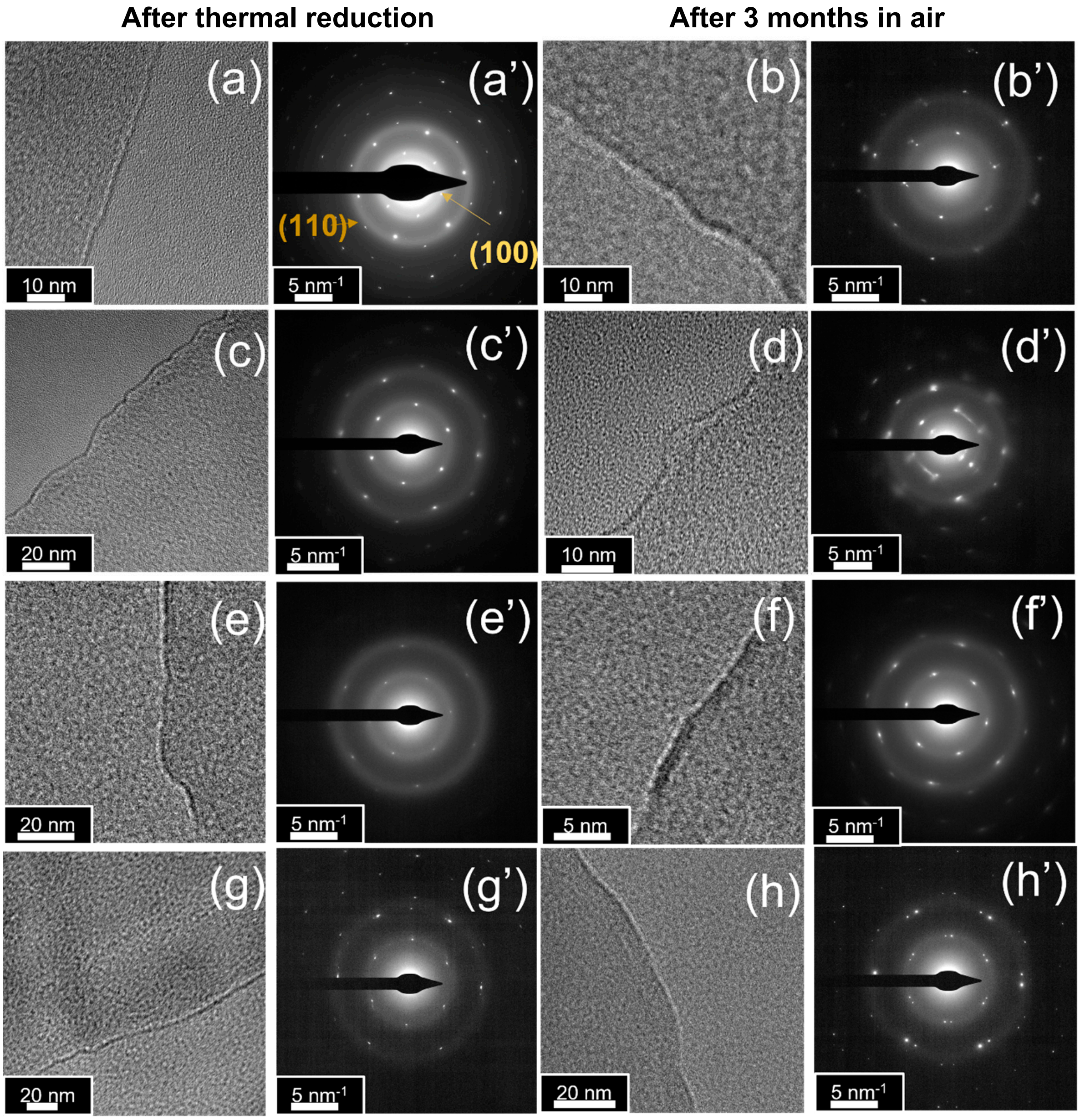

Figure S12. TEM images and respective SAED patterns of REGO after thermal reduction at 900°C and 3 months of storage in air. The samples were prepared using the following electrolytes: (a, a', b, b') 0.1 M H_2SO_4, (c, c', d, d') 0.1 M $(NH_4)_2SO_4$, (e, e', f, f') 0.1 M H_2SO_4 + 1 M HNO_3 50 vol%, (g, g', h, h') 0.1 M $(NH_4)_2SO_4$ + 1 M HNO_3 50 vol%.

The SAED patterns were taken on the edges of the graphene sheets. Diffraction patterns with hexagonal symmetry were found in all samples, as expected. However, the diffuse rings reveal the presence of amorphous structures caused by defects, oxygenated functional groups, and nitrogen atoms within the structure [35,36]. The SAEDs of the fresh REGO samples show single diffraction spots from the (100) and (110) planes [37,38]. This is characteristic of samples composed by mono-to-bilayer graphene sheets. Instead, the diffraction patterns of the 3 months old samples have large or multiple adjacent diffraction spots pointing for layer stacking [39-41].

The d-spacing values related to (100) and (110) planes are ca. 2.2 and 1.5 Å, respectively [37,38]. As reported in Table S4 the values didn't vary significantly after 3 months storage in air.

Table S4. D-spacing calculated from the (100) plane (SAED)

	d-spacing (100) / Å	
	After exfoliation and reduction	After 3 months storage
**0.1 M H_2SO_4**	2.22	2.23
**0.1 M $(NH_4)_2SO_4$**	2.33	2.34
**0.1 M H_2SO_4 + 1 M HNO_3 (50 vol%)**	2.26	2.28
**0.1 M $(NH_4)_2SO_4$ + 1 M HNO_3 (50 vol%)**	2.24	2.26

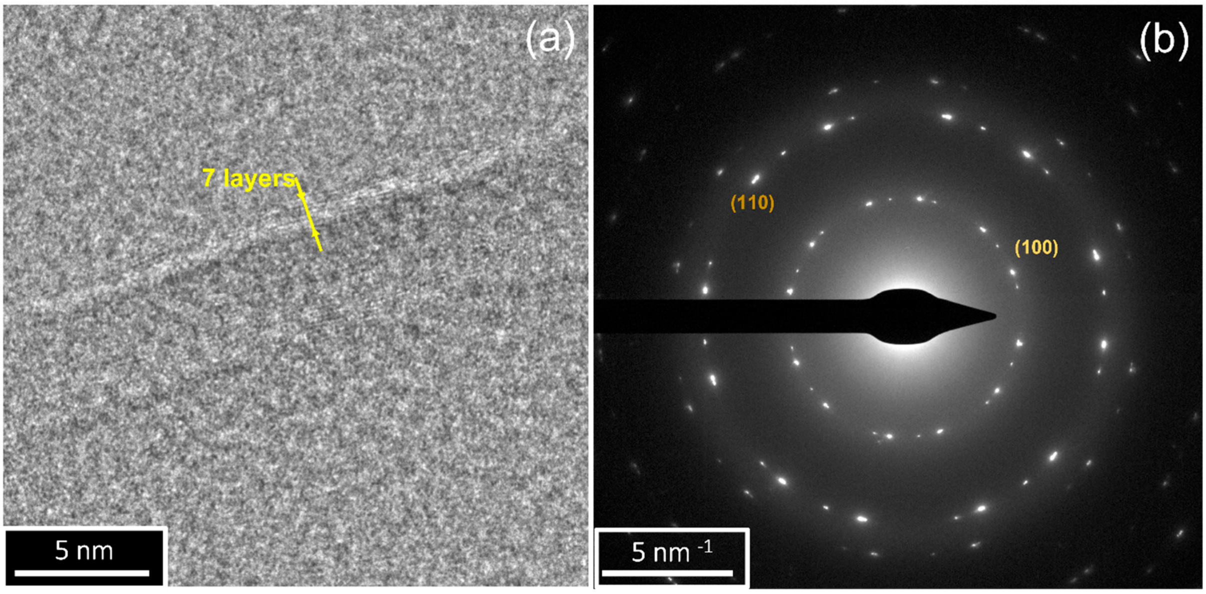

Figure S13. (a) HR-TEM and (b) SAED of REGO synthesized in 1 M HNO_3.

Figure S13 shows an HR-TEM image of REGO synthesized in HNO_3 and its respective SAED pattern. The formation of few layers REGOs is confirmed by HR-TEM where 7 reduced graphene oxide layers REGOs are observed. Additionally, the SAED pattern shows multiple diffraction spots, confirming the presence of multiple layers.

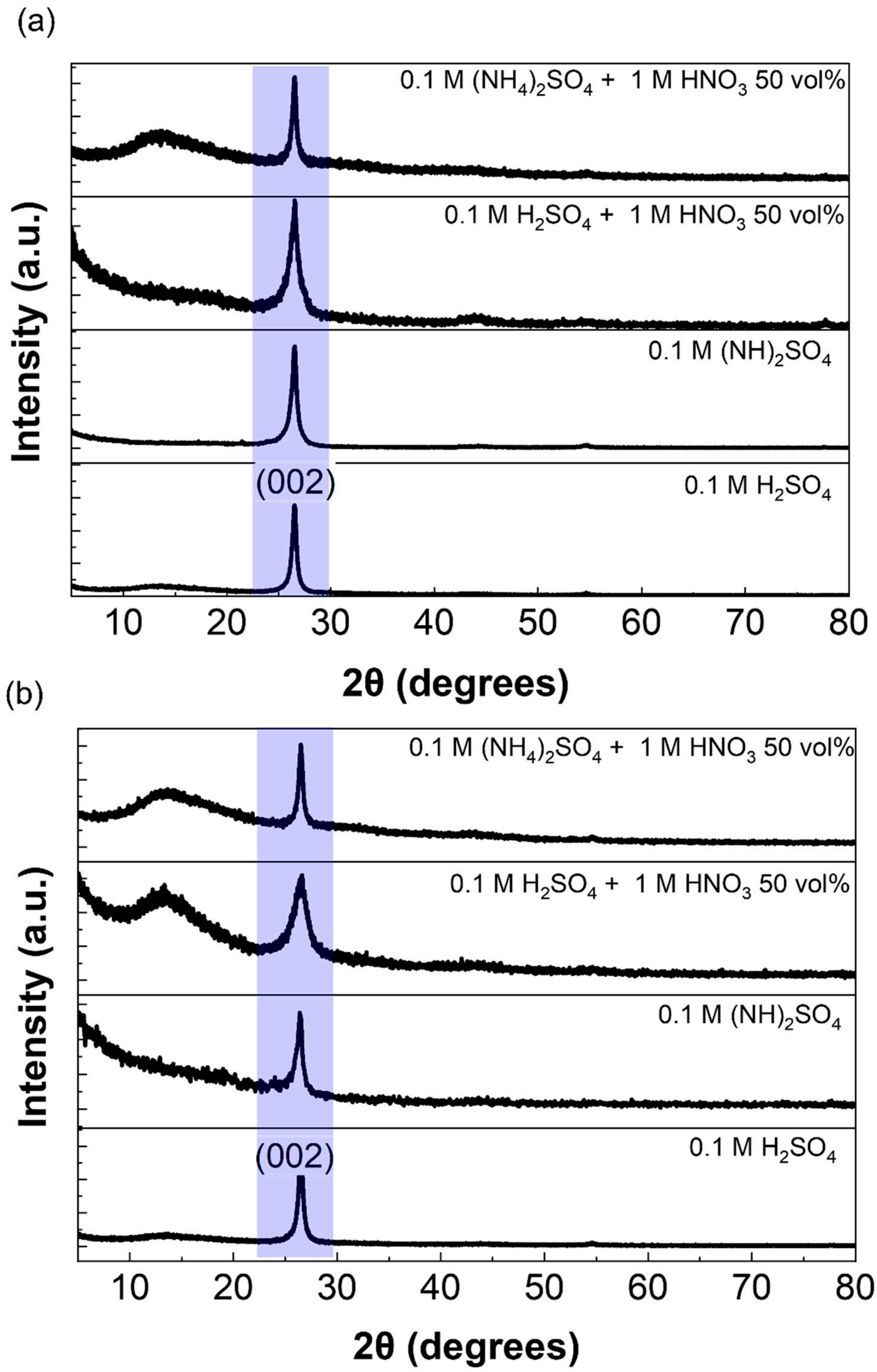

Figure S14. XRD pattern of REGO synthesized in different electrolyte mixtures: (a) after thermal reduction and (b) after 3 months of exposition to air.

The diffraction wave centred at 12 ^{0} is due to the sample holder.

Table S5. D-spacing calculated from the (002) plane (XRD)

	d-spacing (002) / Å	
	**After exfoliation and reduction**	**After 3 months of storage in air**
**0.1 M H_2SO_4**	3.36	3.37
**0.1 M $(NH_4)_2SO_4$**	3.35	3.37
**0.1 M H_2SO_4 + 1 M HNO_3 (50 vol%)**	3.36	3.36
**0.1 M $(NH_4)_2SO_4$ + 1 M HNO_3 (50 vol%)**	3.35	3.36

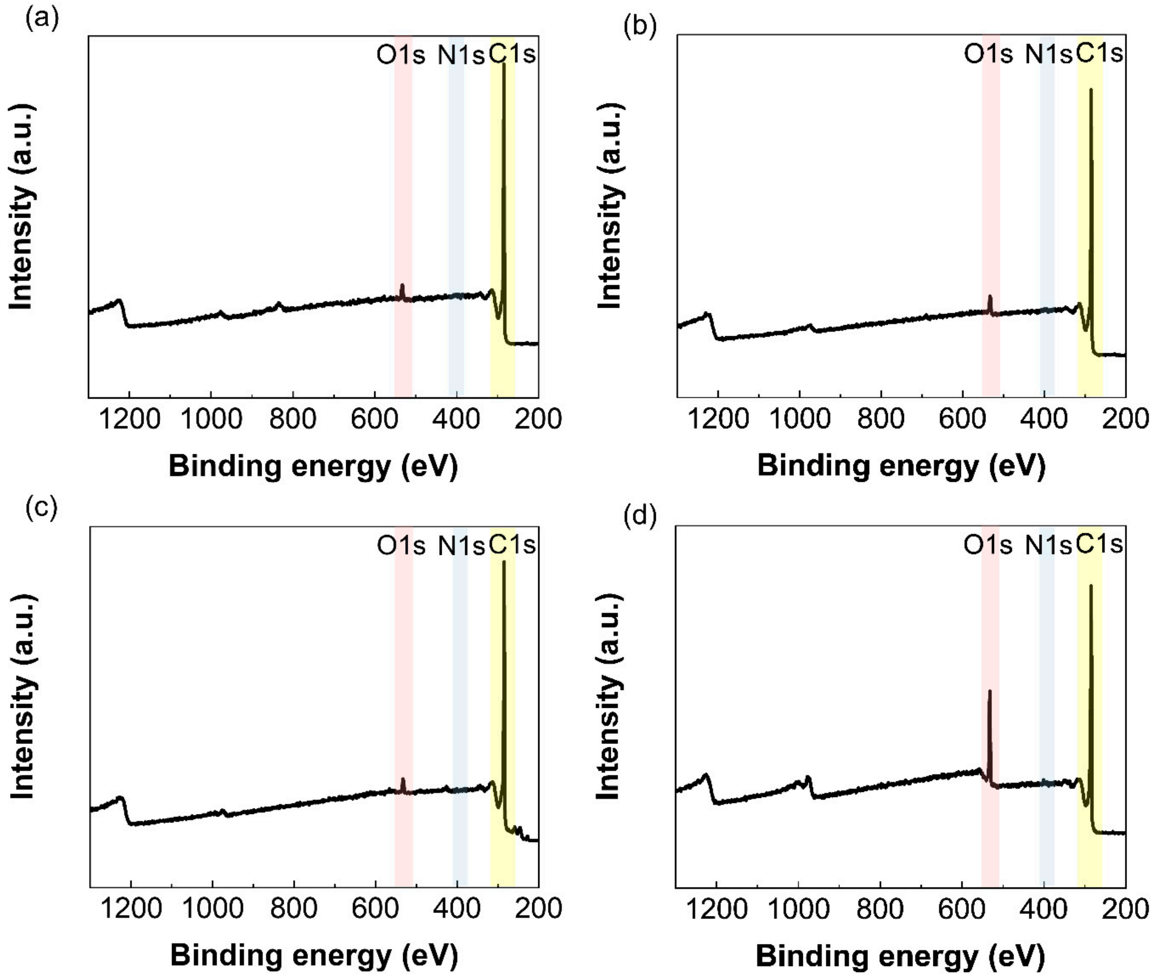

Figure S15. XPS survey spectra of REGO after 3 months storage in air. The samples were prepared using the following electrolytes: (a) 0.1 M $(NH_4)_2SO_4$, (b) 0.1 M $(NH_4)_2SO_4$ + 5 mM HNO_3 (50 vol%), (c) 0.1 M $(NH_4)_2SO_4$ + 1 M HNO_3 (50 vol%), (d) 0.1 M H_2SO_4 + 1 M HNO_3 (50 vol%).

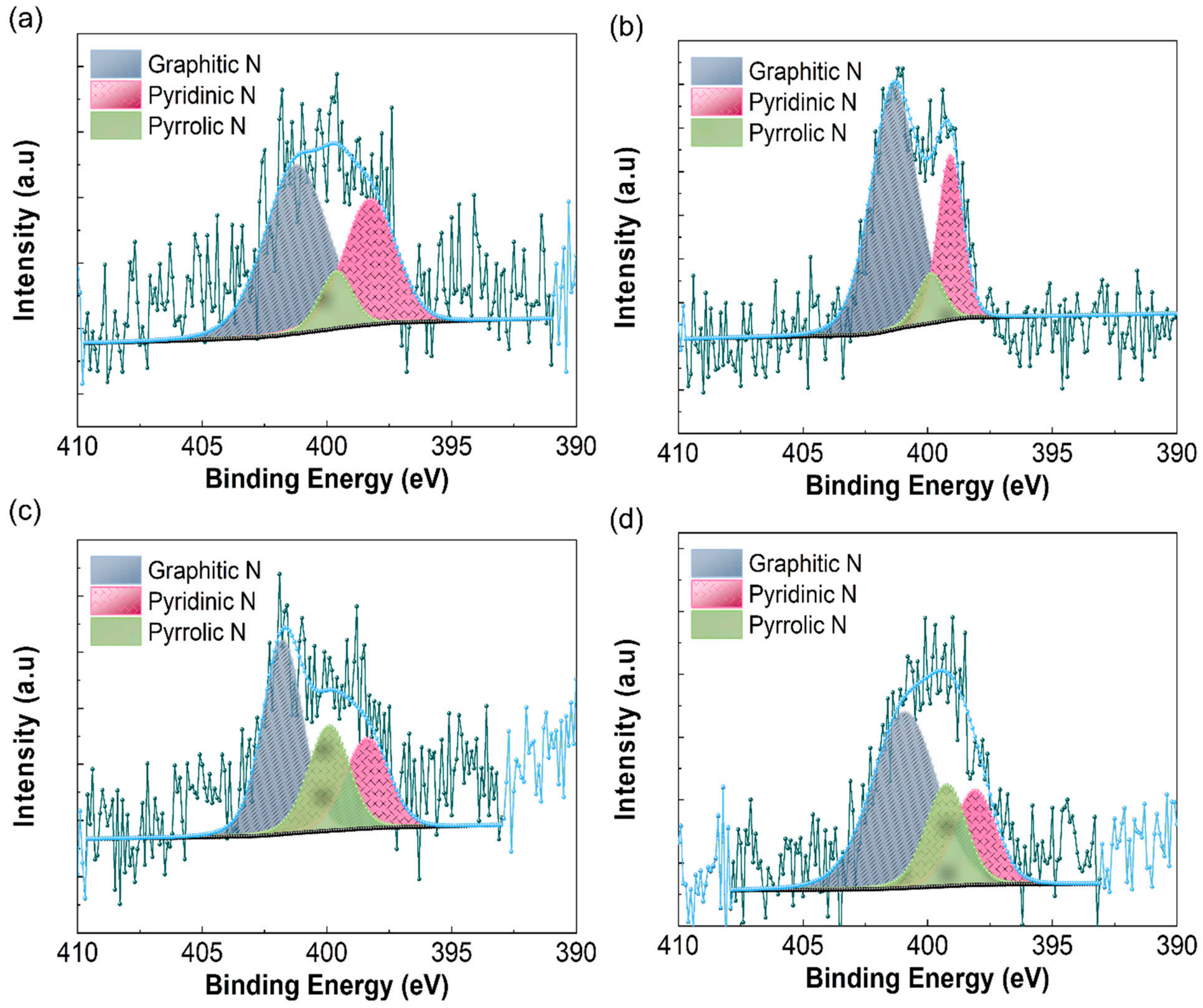

Figure S16. N 1s core level spectra of REGO after 3 months storage in air. The samples were prepared using the following electrolytes: (a) 0.1 M $(NH_4)_2SO_4$, (b) 0.1 M $(NH_4)_2SO_4$ + 5 mM HNO_3 (50 vol%), (c) 0.1 M $(NH_4)_2SO_4$ + 1 M HNO_3 (50 vol%), (d) 0.1 M H_2SO_4 + 1 M HNO_3 (50 vol%).

Table S6. Surface atomic composition after 3 months storage in air

	C at%	O at%	N at%
**$(NH_4)_2SO_4$ 0.1 M**	97.05	2.65	0.30
**$(NH_4)_2SO_4$ 0.1 M + HNO_3 5 mM (50 vol%)**	96.45	3.15	0.40
**$(NH_4)_2SO_4$ 0.1 M + HNO_3 1 M (50 vol%)**	96.56	2.92	0.52
**H_2SO_4 0.1 M + HNO_3 1 M (50 vol%)**	88.06	11.04	0.90

References

1. Ferrari, A.C. Raman spectroscopy of graphene and graphite: Disorder, electron-phonon coupling, doping and nonadiabatic effects. *Solid State Communications* **2007**, *143*, 47-57, doi:10.1016/j.ssc.2007.03.052.
2. Lee, C.; Wei, X.; Kysar, J.W.; Hone, J. Measurement of the elastic properties and intrinsic strength of monolayer graphene. *Science* **2008**, *321*, 385-388, doi:10.1126/science.1157996.
3. Ferrari, A.C.; Meyer, J.C.; Scardaci, V.; Casiraghi, C.; Lazzeri, M.; Mauri, F.; Piscanec, S.; Jiang, D.; Novoselov, K.S.; Roth, S.; et al. Raman spectrum of graphene and graphene layers. *Phys Rev Lett* **2006**, *97*, 187401, doi:10.1103/PhysRevLett.97.187401.
4. Ferrari, A.C.; Robertson, J. Interpretation of Raman spectra of disordered and amorphous carbon. *Physical Review B* **2000**, *61*, 14095-14107, doi:10.1103/PhysRevB.61.14095.
5. Martins Ferreira, E.H.; Moutinho, M.V.O.; Stavale, F.; Lucchese, M.M.; Capaz, R.B.; Achete, C.A.; Jorio, A. Evolution of the Raman spectra from single-, few-, and many-layer graphene with increasing disorder. *Physical Review B* **2010**, *82*, 125429, doi:10.1103/PhysRevB.82.125429.
6. Graf, D.; Molitor, F.; Ensslin, K.; Stampfer, C.; Jungen, A.; Hierold, C.; Wirtz, L. Spatially Resolved Raman Spectroscopy of Single- and Few-Layer Graphene. *Nano letters* **2007**, *7*, 238-242, doi:10.1021/nl061702a.
7. Papanai, G.S.; Sharma, I.; Gupta, B.K. Probing number of layers and quality assessment of mechanically exfoliated graphene via Raman fingerprint. *Materials Today Communications* **2020**, *22*, 100795, doi:10.1016/j.mtcomm.2019.100795.
8. Loh, K.P.; Bao, Q.; Eda, G.; Chhowalla, M. Graphene oxide as a chemically tunable platform for optical applications. *Nat Chem* **2010**, *2*, 1015-1024, doi:10.1038/nchem.907.
9. Ossonon, B.D.; Belanger, D. Synthesis and characterization of sulfophenyl-functionalized reduced graphene oxide sheets. *Rsc Adv* **2017**, *7*, 27224-27234, doi:10.1039/c6ra28311j.

10. Liu, W.; Cao, T.; Dai, X.; Bai, Y.; Lu, X.; Li, F.; Qi, W. Nitrogen-Doped Graphene Monolith Catalysts for Oxidative Dehydrogenation of Propane. *Front Chem* **2021**, *9*, 759936, doi:10.3389/fchem.2021.759936.
11. Yang, D.; Velamakanni, A.; Bozoklu, G.; Park, S.; Stoller, M.; Piner, R.D.; Stankovich, S.; Jung, I.; Field, D.A.; Ventrice, C.A.; et al. Chemical analysis of graphene oxide films after heat and chemical treatments by X-ray photoelectron and Micro-Raman spectroscopy. *Carbon* **2009**, *47*, 145-152, doi:10.1016/j.carbon.2008.09.045.
12. Kim, H.; Namgung, R.; Singha, K.; Oh, I.K.; Kim, W.J. Graphene oxide-polyethylenimine nanoconstruct as a gene delivery vector and bioimaging tool. *Bioconjug Chem* **2011**, *22*, 2558-2567, doi:10.1021/bc200397j.
13. Avila-Vega, Y.I.; Leyva-Porras, C.C.; Mireles, M.; Quevedo-Lopez, M.; Macossay, J.; Bonilla-Cruz, J. Nitroxide-Functionalized Graphene Oxide from Graphite Oxide. *Carbon N Y* **2013**, *63*, 376–389, doi:10.1016/j.carbon.2013.06.093.
14. Baldovino, F.H.; Quitain, A.T.; Dugos, N.P.; Roces, S.A.; Koinuma, M.; Yuasa, M.; Kida, T. Synthesis and characterization of nitrogen-functionalized graphene oxide in high-temperature and high-pressure ammonia. *Rsc Adv* **2016**, *6*, 113924-113932, doi:10.1039/c6ra22885b.
15. Lazar, P.; Mach, R.; Otyepka, M. Spectroscopic Fingerprints of Graphitic, Pyrrolic, Pyridinic, and Chemisorbed Nitrogen in N-Doped Graphene. *J Phys Chem C* **2019**, *123*, 10695-10702, doi:10.1021/acs.jpcc.9b02163.
16. Cui, Z.; Coletta, C.; Dazzi, A.; Lefrancois, P.; Gervais, M.; Neron, S.; Remita, S. Radiolytic method as a novel approach for the synthesis of nanostructured conducting polypyrrole. *Langmuir* **2014**, *30*, 14086-14094, doi:10.1021/la5037844.
17. de Ávila, S.G.; Logli, M.A.; Matos, J.R. Kinetic study of the thermal decomposition of monoethanolamine (MEA), diethanolamine (DEA), triethanolamine (TEA) and methyldiethanolamine (MDEA). *International Journal of Greenhouse Gas Control* **2015**, *42*, 666-671, doi:10.1016/j.ijggc.2015.10.001.

18. Gennadii, M.N.; Georgii, B.M. Thermal decomposition of aliphatic nitro-compounds. *Russian Chemical Reviews* **1994**, *63*, 313, doi:10.1070/RC1994v063n04ABEH000086.

19. Alazmi, A.; Rasul, S.; Patole, S.P.; Costa, P.M.F.J. Comparative study of synthesis and reduction methods for graphene oxide. *Polyhedron* **2016**, *116*, 153-161, doi:10.1016/j.poly.2016.04.044.

20. Peng, J.; Sergiienko, A.; Mangolini, F.; Stallworth, P.E.; Greenbaum, S.; Carpick, R.W. Solid state magnetic resonance investigation of the thermally-induced structural evolution of silicon oxide-doped hydrogenated amorphous carbon. *Carbon* **2016**, *105*, 163-175, doi:10.1016/j.carbon.2016.04.021.

21. Farivar, F.; Yap, P.L.; Hassan, K.; Tung, T.T.; Tran, D.N.H.; Pollard, A.J.; Losic, D. Unlocking thermogravimetric analysis (TGA) in the fight against "Fake graphene" materials. *Carbon* **2021**, *179*, 505-513, doi:10.1016/j.carbon.2021.04.064.

22. Murata, H.; Nakajima, Y.; Saitoh, N.; Yoshizawa, N.; Suemasu, T.; Toko, K. High-Electrical-Conductivity Multilayer Graphene Formed by Layer Exchange with Controlled Thickness and Interlayer. *Scientific Reports* **2019**, *9*, 4068, doi:10.1038/s41598-019-40547-0.

23. Kim, H.W.; Bukas, V.J.; Park, H.; Park, S.; Diederichsen, K.M.; Lim, J.; Cho, Y.H.; Kim, J.; Kim, W.; Han, T.H.; et al. Mechanisms of Two-Electron and Four-Electron Electrochemical Oxygen Reduction Reactions at Nitrogen-Doped Reduced Graphene Oxide. *Acs Catalysis* **2020**, *10*, 852−863, doi:10.1021/acscatal.9b04106.

24. Li, C.; Hu, Y.; Yu, M.H.; Wang, Z.F.; Zhao, W.X.; Liu, P.; Tong, Y.X.; Lu, X.H. Nitrogen doped graphene paper as a highly conductive, and light-weight substrate for flexible supercapacitors. *Rsc Adv* **2014**, *4*, 51878-51883, doi:10.1039/c4ra11024b.

25. Zhang, H.; Kuila, T.; Kim, N.H.; Yu, D.S.; Lee, J.H. Simultaneous reduction, exfoliation, and nitrogen doping of graphene oxide via a hydrothermal reaction for energy storage electrode materials. *Carbon* **2014**, *69*, 66-78, doi:10.1016/j.carbon.2013.11.059.

26. Yang, S.; Ricciardulli, A.G.; Liu, S.; Dong, R.; Lohe, M.R.; Becker, A.; Squillaci, M.A.; Samori, P.; Mullen, K.; Feng, X. Ultrafast Delamination of Graphite into High-Quality Graphene Using Alternating Currents. *Angew Chem Int Ed Engl* **2017**, *56*, 6669-6675, doi:10.1002/anie.201702076.
27. Boas, C.; Focassio, B.; Marinho, E., Jr.; Larrude, D.G.; Salvadori, M.C.; Leao, C.R.; Dos Santos, D.J. Characterization of nitrogen doped grapheme bilayers synthesized by fast, low temperature microwave plasma-enhanced chemical vapour deposition. *Sci Rep* **2019**, *9*, 13715, doi:10.1038/s41598-019-49900-9.
28. Stankovich, S.; Dikin, D.A.; Piner, R.D.; Kohlhaas, K.A.; Kleinhammes, A.; Jia, Y.; Wu, Y.; Nguyen, S.T.; Ruoff, R.S. Synthesis of graphene-based nanosheets via chemical reduction of exfoliated graphite oxide. *Carbon* **2007**, *45*, 1558-1565, doi:10.1016/j.carbon.2007.02.034.
29. Chen, Y.; Fu, K.; Zhu, S.; Luo, W.; Wang, Y.; Li, Y.; Hitz, E.; Yao, Y.; Dai, J.; Wan, J.; et al. Reduced Graphene Oxide Films with Ultrahigh Conductivity as Li-Ion Battery Current Collectors. *Nano letters* **2016**, *16*, 3616-3623, doi:10.1021/acs.nanolett.6b00743.
30. Zhang, W.W.; Luo, Q.P.; Duan, X.H.; Zhou, Y.; Pei, C.H. Nitrated graphene oxide and its catalytic activity in thermal decomposition of ammonium perchlorate. *Materials Research Bulletin* **2014**, *50*, 73-78, doi:10.1016/j.materresbull.2013.10.023.
31. Zhang, X.; Wang, Y.; Wang, Y.; Guo, Y.; Xie, X.; Yu, Y.; Zhang, B. Recent advances in electrocatalytic nitrite reduction. *Chem Commun (Camb)* **2022**, *58*, 2777-2787, doi:10.1039/d1cc06690k.
32. Yun, Y.S.; Yoon, G.; Park, M.; Cho, S.Y.; Lim, H.-D.; Kim, H.; Park, Y.W.; Kim, B.H.; Kang, K.; Jin, H.-J. Restoration of thermally reduced graphene oxide by atomic-level selenium doping. *NPG Asia Materials* **2016**, *8*, e338-e338, doi:10.1038/am.2016.191.
33. Park, H.; Lim, S.; Nguyen, D.D.; Suk, J.W. Electrical Measurements of Thermally Reduced Graphene Oxide Powders under Pressure. *Nanomaterials-Basel* **2019**, *9*, 1387.
34. Er, E.; Çelikkan, H. An efficient way to reduce graphene oxide by water elimination using phosphoric acid. *Rsc Adv* **2014**, *4*, 29173-29179, doi:10.1039/C4RA03204G.

35. Lv, Z.; Wang, Z.; Chen, J. Nitrogen doped small molecular structures of nano-graphene for high-performance anodes suitable for lithium ion storage. *Rsc Adv* **2019**, *9*, 22401-22409, doi:10.1039/c9ra02498k.
36. Ngidi, N.P.D.; Ollengo, M.A.; Nyamori, V.O. Effect of Doping Temperatures and Nitrogen Precursors on the Physicochemical, Optical, and Electrical Conductivity Properties of Nitrogen-Doped Reduced Graphene Oxide. *Materials* **2019**, *12*, 3376, doi:10.3390/ma12203376.
37. Wang, G.; Yang, J.; Park, J.; Gou, X.; Wang, B.; Liu, H.; Yao, J. Facile Synthesis and Characterization of Graphene Nanosheets. *The Journal of Physical Chemistry C* **2008**, *112*, 8192-8195, doi:10.1021/jp710931h.
38. Zheng, B.; Chen, Y.; Li, P.; Wang, Z.; Cao, B.; Qi, F.; Liu, J.; Qiu, Z.; Zhang, W. Ultrafast ammonia-driven, microwave-assisted synthesis of nitrogen-doped graphene quantum dots and their optical properties. *Nanophotonics* **2017**, *6*, 259-267, doi:10.1515/nanoph-2016-0102.
39. Feng, S.; dos Santos, M.C.; Carvalho, B.R.; Lv, R.; Li, Q.; Fujisawa, K.; Elías, A.L.; Lei, Y.; Perea-López, N.; Endo, M.; et al. Ultrasensitive molecular sensor using N-doped graphene through enhanced Raman scattering. *Science Advances* **2016**, *2*, e1600322, doi:doi:10.1126/sciadv.1600322.
40. Parvez, K.; Li, R.; Puniredd, S.R.; Hernandez, Y.; Hinkel, F.; Wang, S.; Feng, X.; Mullen, K. Electrochemically exfoliated graphene as solution-processable, highly conductive electrodes for organic electronics. *ACS nano* **2013**, *7*, 3598-3606, doi:10.1021/nn400576v.
41. Yan, Z.; Peng, Z.; Sun, Z.; Yao, J.; Zhu, Y.; Liu, Z.; Ajayan, P.M.; Tour, J.M. Growth of Bilayer Graphene on Insulating Substrates. *ACS nano* **2011**, *5*, 8187-8192, doi:10.1021/nn202829y.
